# Pathway-based subnetworks enable cross-disease biomarker discovery

**DOI:** 10.1038/s41467-018-07021-3

**Published:** 2018-11-12

**Authors:** Syed Haider, Cindy Q. Yao, Vicky S. Sabine, Michal Grzadkowski, Vincent Stimper, Maud H. W. Starmans, Jianxin Wang, Francis Nguyen, Nathalie C. Moon, Xihui Lin, Camilla Drake, Cheryl A. Crozier, Cassandra L. Brookes, Cornelis J. H. van de Velde, Annette Hasenburg, Dirk G. Kieback, Christos J. Markopoulos, Luc Y. Dirix, Caroline Seynaeve, Daniel W. Rea, Arek Kasprzyk, Philippe Lambin, Pietro Lio’, John M. S. Bartlett, Paul C. Boutros

**Affiliations:** 1 Informatics and Biocomputing Program, Ontario Institute for Cancer Research, Toronto, M5G 0A3 Canada; 2 Computer Laboratory, University of Cambridge, Cambridge, CB3 0FD United Kingdom; 3 Diagnostic Development Program, Ontario Institute for Cancer Research, Toronto, M5G 0A3 Canada; 4 Department of Medical Biophysics, University of Toronto, Toronto, M5G 1L7 Canada; 5 Department of Radiation Oncology (Maastro), GROW-School for Oncology and Developmental Biology, Maastricht University Medical Center, Maastricht, The Netherlands; 6 Cancer Research UK Clinical Trials Unit, University of Birmingham, Birmingham, B15 2TT United Kingdom; 7 Leiden University Medical Center, Leiden, The Netherlands; 8 University Hospital, Freiburg, Germany; 9 Klinikum Vest Medical Center, Marl, Germany; 10 Athens University Medical School, Athens, Greece; 11 St. Augustinus Hospital, Antwerp, Belgium; 12 Erasmus Medical Center-Daniel den Hoed, Rotterdam, The Netherlands; 13 Department of Pharmacology and Toxicology, University of Toronto, Toronto, M5S 1A8 Canada

## Abstract

Biomarkers lie at the heart of precision medicine. Surprisingly, while rapid genomic profiling is becoming ubiquitous, the development of biomarkers usually involves the application of bespoke techniques that cannot be directly applied to other datasets. There is an urgent need for a systematic methodology to create biologically-interpretable molecular models that robustly predict key phenotypes. Here we present SIMMS (Subnetwork Integration for Multi-Modal Signatures): an algorithm that fragments pathways into functional modules and uses these to predict phenotypes. We apply SIMMS to multiple data types across five diseases, and in each it reproducibly identifies known and novel subtypes, and makes superior predictions to the best bespoke approaches. To demonstrate its ability on a new dataset, we profile 33 genes/nodes of the PI3K pathway in 1734 FFPE breast tumors and create a four-subnetwork prediction model. This model out-performs a clinically-validated molecular test in an independent cohort of 1742 patients. SIMMS is generic and enables systematic data integration for robust biomarker discovery.

## Abstract (web summary)

Accurate and actionable biomarkers that integrate diverse molecular, functional and clinical information hold great promise in precision medicine. Here, the authors develop SIMMS, a method for pathway-based cross-disease biomarker discovery.

## Introduction

Most human disease is complex, caused by interaction of genetic, epigenetic and environmental insults. A single disease phenotype can often arise in many ways, allowing a diversity of molecular underpinnings to yield a smaller number of phenotypic consequences. This molecular heterogeneity within a single disease is believed to underlie poor clinical trial results for some therapies^1^ and the modest performance of many genome-wide association studies^2–4^.

Researchers thus face two challenges. First, molecular markers are needed to personalize and optimize treatment decisions by predicting patient outcome (prognosis/residual risk) and response to therapy. Second, clinical heterogeneity in patient phenotypes needs to be molecularly rationalized to allow targeting of the mechanistic underpinnings of disease. For example, if a single pathway is dysregulated in multiple ways, drugs targeting the pathway could be applied.

Several approaches have been taken to solve these challenges. The most common has been to measure mRNA abundances as a snapshot of cellular state, and to construct a predictive model from them^5,6^. Unfortunately, these studies have been limited by noise and disease heterogeneity. Several groups have integrated multiple data types using network and systems biology approaches identifying patient subtypes, with limited post-hoc clinical evaluation^7–25^. These algorithms have not yet clearly shown how the interplay between different pathways underpins disease etiology, nor generated biomarkers with systematically demonstrated reproducibility on independent patient cohorts across multiple indications^26^.

There is thus an urgent need to generate accurate and actionable biomarkers that integrate diverse molecular, functional and clinical information. We developed a subnetwork-based approach, called SIMMS, which uses arbitrary molecular data types to identify dysregulated pathways and create functional biomarkers. We validate SIMMS across five tumor types and 11,392 patients, using it to create biomarkers from a diverse range of molecular assays and uncovering unanticipated pan-cancer similarities.

## Results

### Prioritization of candidate prognostic subnetworks

SIMMS acts upon a collection of subnetwork modules, where each node is a molecule (e.g., a gene or metabolite) and each edge is an interaction (physical or functional) between those molecules. Molecular data is projected onto these subnetworks using topology measurements that represent the impact of and synergy between different molecular features. To allow modeling of biological processes with different network architectures, we devised three scoring paradigms: N (nodes/molecules in a subnetwork), E (edges/interactions in a subnetwork) and N + E (both nodes and edges). While the N model assumes independent and additive effects of parts of a subnetwork, the E and N + E models incorporate the impact of dysregulated interactions (Methods). SIMMS fits each one of these models thereby estimating a ‘module-dysregulation score’ (MDS) for each subnetwork that measures their strength of association with a specific disease, phenotype or outcome (Supplementary Fig. 1).

### Characteristics and benchmarking of prognostic subnetworks

A key challenge faced by translational research is to extend the single gene biomarkers paradigm to clinically actionable metagenes/pathways. Thus, we tested the prognostic value of pathway-derived subnetworks using Cox modeling to quantify how effectively a subnetwork stratifies patients into groups with differential risk (Methods). SIMMS can use any network, and we chose to evaluate it using 449 gene-centric pathways from the high-quality, manually-curated NCI-Nature Pathway Interaction database^27^. For each pathway, interconnected proteins (protein-protein interactions or protein complexes) were isolated and regarded as a subnetwork. We further removed overlapping subnetworks from this collection resulting in 500 subnetworks across the database (Supplementary Table 1; Supplementary Fig. 2; Methods section: Pathways database pre-processing). We then trained and tested SIMMS on a series of large and well-curated mRNA abundance datasets of primary breast (1010 training patients; 1098 validation patients), colon (205 training; 439 validation), lung (380 training; 369 validation) and ovarian (438 training; 566 validation) cancers (Supplementary Tables 2–5; Supplementary Fig. 3; Supplementary Methods section 1).

Our analysis of prognostic subnetworks revealed several properties of tumor network biology. First, there was a global propensity for highly prognostic subnetworks to contain significantly higher number of genes and interactions for Model N and N + E (*P* < 0.05, Wilcoxon rank sum test; Supplementary Fig. 4). This association between subnetwork size (number of genes) and prognostic ability was consistent in breast, NSCLC and ovarian cancers, even though different pathways were altered in each but not in colon cancers. Second, the prognostic ability of Model N was significantly superior to that of Model N + E and E; a trend which was maintained across all four cancer types (one-way ANOVA, Tukey HSD multiple comparison test; Supplementary Fig. 5**)**. This suggests that mRNA abundance of functionally-related genesets alone is a strong predictor of patient outcome; here a geneset refers to a set of genes from the same subnetwork. We therefore focused solely on Model N moving forward, while recognizing that in other diseases different subnetwork architectures may be disrupted and therefore require model E or N + E.

**Fig. 1:**
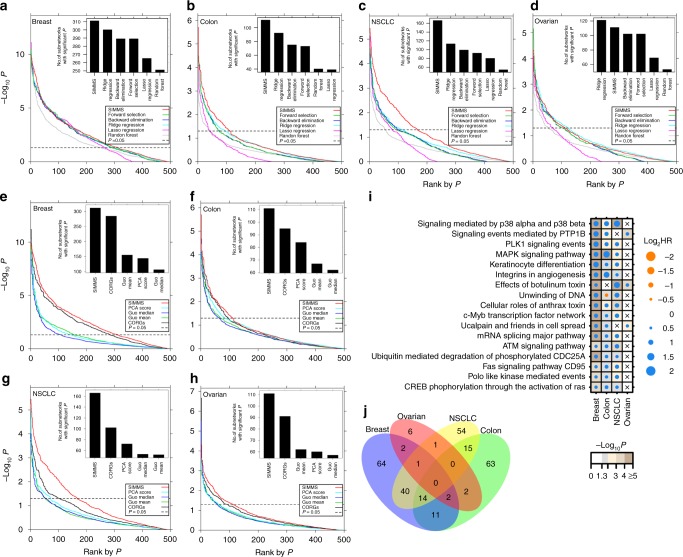
Benchmarking prognostic subnetworks. **a** Comparison of prognostic ability of subnetworks in validation sets of breast cancer using SIMMS and five machine learning algorithms. For each algorithm, Wald *P* values were ranked in increasing order. The number of validated subnetworks identified by each algorithm (*P* < 0.05, above horizontal dashed line) are shown as barplots. **b**–**d** Same visualization as (**a**) using data for colon, NSCLC and ovarian cancers. **e** Comparison of SIMMS against other pathway/subnetwork scoring methods. For each method, ranked P values and total number of significant subnetworks are shown following prognostic assessment in breast cancer validation sets. **f–h** Same as (**e**) using data for colon, NSCLC and ovarian cancers. **i** Dot plot of univariate hazard ratios and *P* values (Wald-test) for each of the top *n* subnetworks significantly associated with patient outcome (|log_2_ HR| > 0.584, *P* < 0.05) in at least 3/4 cancer types. A Cox proportional hazards model was fitted to dichotomized risk scores across the entire validation cohort. Crosses represent absence of a module from a particular cancer type. **j** Overlap of candidate subnetwork markers across breast, colon, NSCLC and ovarian cancers

Next we compared how SIMMS subnetwork scores perform against five well-known machine learning algorithms treating genes as individual features in multivariate setting (Supplementary Methods section 2). SIMMS identified an equal or significantly greater number of prognostic subnetworks compared to models based on genes in each of these subnetworks for these methods (Comparison of proportion of significant subnetworks identified by SIMMS vs. other algorithms: *P* < 0.01, proportion test; Fig. 1a-d). We further compared SIMMS against a panel of pathway/subnetwork scoring methods^18,28–30^, each representing a distinct class of summary estimates (Supplementary Methods section 2). SIMMS outperformed all methods identifying significantly higher number of prognostic subnetworks (*P* < 0.05, proportion test; Fig. 1e-h) with an exception of CORGs where SIMMS identified a greater number of subnetworks in all cancer types, however not significant in breast, colon and ovarian cancers (*P* = 0.05–0.17, proportion test). Sensitivity of these methods against a panel of subnetworks most likely to be associated with patient outcome (having at least three significantly prognostic genes) also confirmed SIMMS’ superiority with highest true positive rate compared to other methods across all four cancer types (Supplementary Fig. 6).

### Multi-cancer prognostic subnetworks

We next quantitatively determined the similarity between different tumor types at the pathway level. Cross disease assessment of significantly prognostic subnetworks (Wald *P* < 0.05) revealed well-known oncogenic pathways such as Aurora Kinase A and B signaling, apoptosis, DNA repair, *RAS* signaling, telomerase regulation and *P53* activity in all four tumor types (Supplementary Tables 6–9). By limiting to highly prognostic subnetworks (|log_2_HR| > 0.584 and *P* < 0.05) in each tumor type, 17 recurrently prognostic subnetworks (at least three tumor types) were identified (Fig. 1i, Supplementary Fig. 7). Significant overlap between prognostic subnetworks was observed for breast, colon, and NSCLC (14 subnetworks: *P*_overlap_ = 0.009, 10^5^ permutations; Fig. 1j), thereby highlighting repurposing potential of anti-cancer drugs targeting these subnetworks. We further assessed whether mRNA dysregulation in these prognostic subnetworks is simply a read out of somatic mutations acquired in the underlying genes. Prognostic assessment of mutation burden in TCGA datasets for these subnetworks revealed only two as prognostic; one in breast and one in lung cancer (Supplementary Fig. 8).

As chemo-resistance is a critical unmet need for cancer patients, we tested prognostic subnetworks for predictive potential. We used TCGA ovarian cancer platinum response data and tested for enrichment of responders and non-responders in SIMMS’ predicted risk groups for each of the significantly prognostic subnetworks (Wald *P* < 0.05). In total, 7/111 subnetworks demonstrated co-occurrence of platinum resistance in high-risk groups (Odds ratio > 2, *P* < 0.05, Supplementary Table S9d). Most of the genes underlying these subnetworks are involved in *Keratinocyte differentiation*, *p38 MAPK signaling* and *Regulation of telomerase* (Supplementary Fig. 9). These pathways require further validation beyond this correlative potential, however these data show the potential of SIMMS for development of predictive, as well as prognostic, biomarkers.

**Fig. 2:**
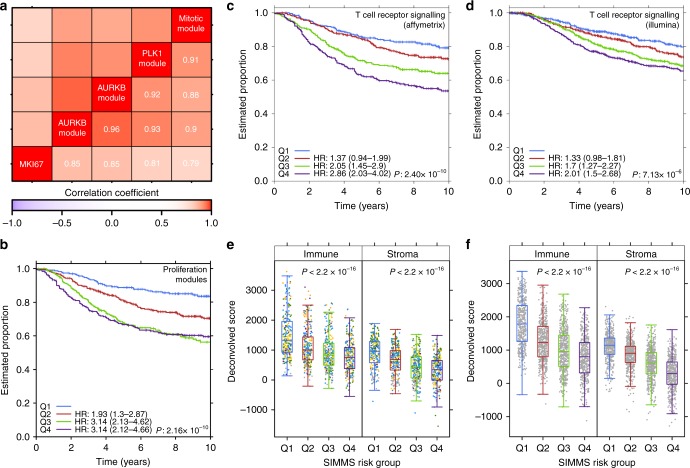
Proliferation and immuno subnetworks. **a** Heatmap of correlation (Spearman) and cluster analysis of patient’s risk scores of proliferation modules in breast cancer, alongside mRNA abundance of a proliferation marker *MKI67*. Ward’s method was used for hierarchical clustering. Data shown for validation cohorts. **b** Kaplan–Meier analysis of predicted proliferation scores (validation cohorts) using SIMMS-derived proliferation biomarker. Groups (Q1-Q4) were established using quartiles derived from the training set. Groups Q2-Q4 were compared to Q1 using Cox proportional hazards model. *P* value was estimated using Log-rank test assessing heterogeneity across the four groups. **c** Kaplan–Meier analysis of tumor immune microenvironment driver subnetwork (BioCarta pathway: T cell receptor signaling) in Affymetrix based validation cohorts. Quartile based risk groups (thresholds derived from training set), demonstrating linear increase in the likelihood of recurrence/event. Test statistics same as in **b**. **d** Kaplan–Meier analysis of tumor immune microenvironment driver subnetwork (BioCarta pathway: T cell receptor signaling) in Metabric breast cancer cohort (Illumina platform). **e** Assessment of computationally inferred immune system infiltration and stromal estimates against SIMMS predicted risk groups (Q1-Q4 i.e., low to high) in Affymetrix validation cohorts (test statistic: ANOVA *P* value). Color of dots represent respective validation cohort (Supplementary Table 2). **f** Same as **e** using Metabric cohort (Illumina platform)

In breast cancer, subnetwork modules encompassing proliferation pathways (Mitosis, *PLK1*, *AURKA,* and *AURKB*) were highly prognostic (Supplementary Table 6b). To ensure these are not driven by common proliferation genes, we tested gene overlap in these subnetworks and found them highly divergent (Supplementary Fig. 10a). We further tested whether estimated risk scores of these four subnetwork modules recapitulate proliferation accurately. We used the *MKI67* (mRNA abundance) as a surrogate for proliferation, and found strong concordance between *MKI67* abundance and SIMMS’ risk scores (Spearman’s *ρ* = 0.79–0.86, *P* < 10^–3^; Fig. 2a). To determine if subnetworks more accurately model patient-relevant biology, we constructed a multivariate proliferation signature using the four modules. This signature was a robust prognostic marker (Fig. 2b) and presents an opportunity to understand the functionally-related proliferation correlates of patient outcome beyond single gene markers. We next investigated prognostic subnetworks focusing on clinically actionable pathways. In breast cancer, immune microenvironment subnetwork of *T cell receptor signaling* was a significant predictor of patient outcome (HR_Q1-Q4_ = 2.86, 95% CI = 2.03–4.02, *P* = 1.78 × 10^–9^; Fig. 2c, Supplementary Table 6d), in particular, distant metastasis free survival where data was available (Sotiriou: HR = 3.52, 95% CI = 1.38–9.02, *P* = 0.0086; Wang: HR = 1.58, 95% CI = 1.07–2.33, *P* = 0.02). We further validated this subnetwork for breast cancer disease-specific survival in an independent cohort of 1970 patients^31^ (HR_Q1-Q4_ = 2.01, 95% CI = 1.5–2.68, *P* = 2.41 × 10^–6^; Fig. 2d). Hypothesizing that this subnetwork may serve as a marker of tumor immune infiltration, we confirmed association between SIMMS predicted risk groups and immune cell content^32^ (Affymetrix: Spearman’s *ρ* = −0.38, *P* < 2.2 × 10^–16^; Illumina: Spearman’s *ρ* = −0.48, *P* < 2.2 × 10^–16^), as well as stromal signal (Affymetrix: Spearman’s *ρ* = −0.43, *P* < 2.2 × 10^–16^; Illumina: Spearman’s *ρ* = −0.59, *P* < 2.2 × 10^−16^) (Fig. 2e-f), both of which were associated with good outcome. Consistent with a recent breast cancer study^33^, naïve immune and stromal content estimates were only weakly associated with patient outcome (Supplementary Fig. 10b-e), whilst SIMMS’ MDS of *T-cell receptor signaling* not only provides accurate identification of patients who may benefit from immunotherapy, but also indicates associated molecular targets.

### Multi-subnetwork biomarkers predict patient outcome

**Fig. 3:**
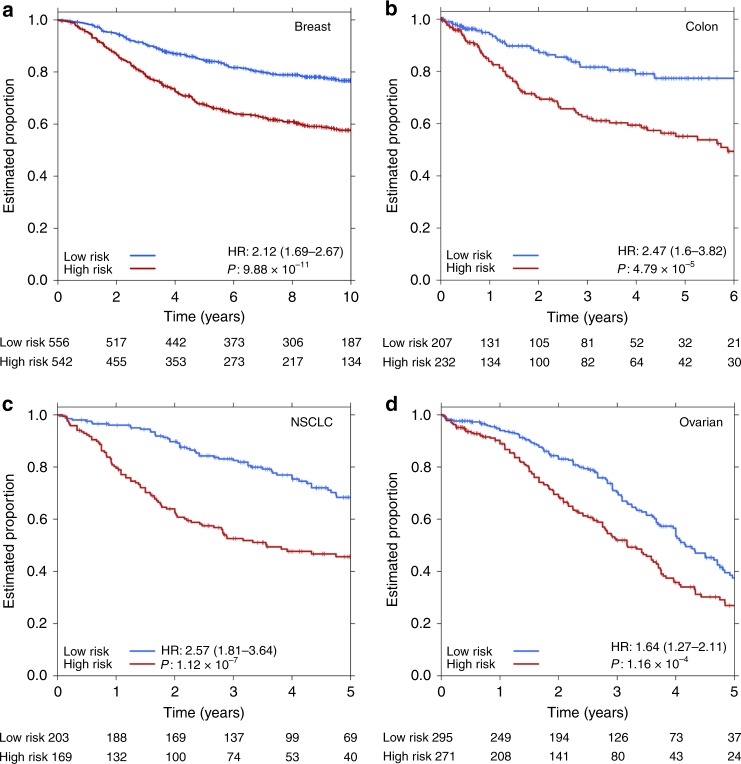
Multi-subnetwork biomarkers for multiple cancer types. **a**–**d** Kaplan–Meier survival plots using Model N over the entire validation cohort with subnetwork selection performed through Cox model using generalized linear models (*L1*-regularization) on the training cohort. Final model resulted in 23/50, 5/75, 23/25, and 23/50 subnetworks for breast, colon, NSCLC and ovarian cancers, respectively (Supplementary Tables 10–13). *P* values were estimated using Wald-test

As SIMMS accurately identified univariate prognostic subnetworks, we hypothesized that modeling of multiple aspects of tumor biology through these subnetworks into a single molecular biomarker could better rationalize patient heterogeneity emerging from alternative pathways of disease progression. First, to initialize the number of subnetworks, 10 million random sets of subnetworks of different sizes (1 to 250) were generated regardless of subnetwork size (Supplementary Fig. 11). These were tested for prognostic potential in a multivariate Cox model, thereby generating an empirical null distribution which allowed us to select the optimal number of subnetworks that influence survival in each disease, as well as to circumvent potential bias towards large subnetworks. Using the optimal number of subnetworks maximizing performance in the training set (n_Breast = _50, n_Colon = _75, n_NSCLC = _25 and n_Ovarian = _50), SIMMS’ risk scores were estimated in each disease. These subnetworks revealed a number of highly correlated clusters of subnetworks (Supplementary Fig. 12–15**)**. Next, multivariate prognostic classifiers (Cox model with *L1-*regularization; 10-fold cross validation) were created for each tumor type thereby further refining the list of highly correlated subnetworks. For each tumor type, subnetwork-based classifiers encompassing multiple pathways successfully predicted patient survival in the fully-independent validation cohorts (Fig. 3, Supplementary Tables 10–13). We verified that these results are not driven by a single cohort or patient subset, but rather reproducible across studies (Supplementary Fig. 16–19). Similarly SIMMS generated robust biomarkers for each tumor-type using multiple feature-selection approaches: multivariate analysis using both backward and forward refinements yielded similar results (Supplementary Fig. 20).

Next, we challenged SIMMS’ modeling of pathway-based features against: (1) a biomarker constructed from all the genes (in our pathways database) in multivariate setting using a Cox model with *L1-*regularization, and (2) a biomarker constructed using all these genes collapsed into one composite geneset which was subsequently modeled using SIMMS. For the large multivariate model, breast and ovarian cancer models yielded results similar to SIMMS while colon and NSCLC models were significantly inferior to SIMMS’ models (Supplementary Fig. 21a-d). Further, modeling the composite geneset using SIMMS improved the performance for colon and NSCLC markers (Supplementary Fig. 21e-h). These, alongside SIMMS’ native models (Fig. 3) highlight a potential saturation point where large models may not yield improved prognostic markers. To ensure SIMMS-derived prognostic markers performed comparably to existing transcriptomic prognostic tools, we compared our four SIMMS prognostic markers to 21 independent approaches using the same test datasets. For each disease, the SIMMS signature performed as-well or better than the best published signature, each of which used a unique methodology (Supplementary Methods section 3, Supplementary Table 14). Therefore SIMMS provided a consistent and unified approach to generating highly accurate biomarkers.

**Fig. 4:**
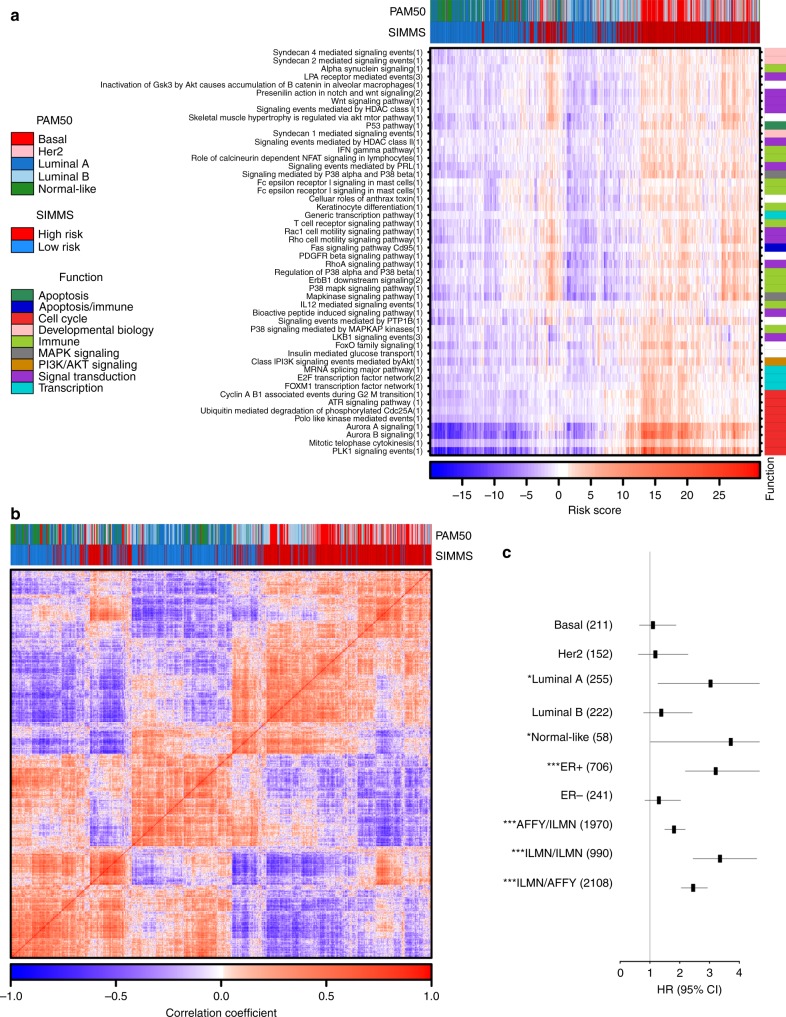
Clinical association of breast cancer biomarkers. **a** Heatmap of patients’ risk scores estimated using top n_Breast=_50 subnetworks in the Metabric validation cohort. Column covariates show patient classifications based on PAM50-based molecular subtypes and SIMMS predicted risk groups. Row covariates indicate functional class of subnetwork’s originating pathway. Columns and rows were clustered using divisive clustering. Number in parenthesis of y-axis labels represents subnetwork number from a given pathway; with details in subnetwork database (SIMMS R package). ‘*Fc Epsilon Receptor I Signaling in Mast Cells*’ is repeated twice because it is represented by two different pathways in the database (ID = 100165 and ID = 200003 in subnetworks database; SIMMS R package). **b** Clustered (divisive) heatmap of correlation (Spearman) between patients using their subnetwork risk score profiles (top n_Breast=_50 subnetworks) in the Metabric validation cohort with covariates as detailed in **a**. **c** Forest plot showing HR and 95% CI (multivariate Cox proportional hazards model) of the breast cancer subtype-specific markers, as well as cross-platform validation. Datasets originating from Illumina (ILMN) and Affymetrix (AFFY) were used in turn for cross platform training and validation. Due to limited availability of clinical annotations on Affymetrix based cohorts, only the Illumina dataset (Metabric) was used for subtype-specific models. For these, the Metabric-published training and validation cohorts were maintained for training and validation purposes. Numbers in parenthesis indicate the size of the validation cohort. Asterisks represent statistical significance of differential outcome between the predicted low-risk and high-risk groups (*P < 0.05, **P < 0.01, ***P < 0.001, Wald-test)

Focusing on breast cancer as a disease with well-defined molecular subtypes, we tested SIMMS on Metabric breast cancer cohort (*n* = 1,970)^31^. Our prognostic classifier revealed two primary patient clusters with distinct pathway activities encompassing subnetworks derived from cell cycle, signaling, immune and regulatory pathways (Fig. 4a, b). These clusters were highly concordant with the PAM50^34^ intrinsic subtypes of breast cancer (*f*-measure = 0.81). Since breast cancer is a heterogeneous disease with distinct molecular and clinical characteristics^34^, we asked whether SIMMS could identify subtype-specific prognostic markers. To evaluate this, we classified patients into PAM50, ER+ and ER− subtypes and created SIMMS classifiers for each subtype. SIMMS classifiers were able to identify subgroups of patients at a significantly higher risk of relapse (Wald *P* < 0.05) in each of the Luminal-A, Normal-like and ER+ subtypes (Fig. 4c). Importantly, these subgroups of patients present differential pathway activity (as quantified by SIMMS), and hence may benefit from aggressive/alternative treatments targetting these pathways. We further validated the efficacy of SIMMS when trained and tested for reproducibulity across different genomic platforms (Affymetrix and Illumina; *P* < 10^-5^; Fig. 4c **AFFY/ILMN, ILMN/ILMN, ILMN/AFFY;** Supplementary Fig. 22). Taken together these results demonstrate that pathway-driven subnetwork modeling can flexibly integrate diverse assays emerging from multiple platforms.

### A PIK3CA signaling risk predictor in early breast cancer

While the public data used to evaluate SIMMS is valuable, it does not closely represent that used in clinical settings. To better represent this scenario, we focused on the PI3K-signaling pathway, which is frequently mutated in breast cancer and is the subject of several targeted therapies. We evaluated 1,734 samples from the phase III TEAM clinical trial and measured mRNA abundance of 33 PI3K signaling genes in clinically-relevant FFPE samples. All samples were ER positive “luminal” breast cancers from the TEAM pathology study^35^ (Supplementary Table 15, Supplementary Methods section 4). We hypothesized that inclusion of key signaling nodes from driver molecular pathways in residual risk signatures would both improve risk stratification and identify candidate theranostic targets for the next generation of clinical trials. Univariate prognostic assessment of 33 genes revealed significant association between seven genes and distant metastasis (Wald *P*_adjusted_ < 0.05; Supplementary Table 16). Survival analysis of clinical covariates indicated tumor grade, N-stage, T-stage and HER2 IHC as predictors of distant metastasis (Supplementary Table 17). Next, we aggregated 33 PI3K signaling genes into 8 functional modules representing different nodes of the pathway (Supplementary Fig. 23, Supplementary Table 18), and applied SIMMS to train a residual risk model. The SIMMS-derived model comprised of four modules and two clinical covariates (Supplementary Table 19).

**Fig. 5:**
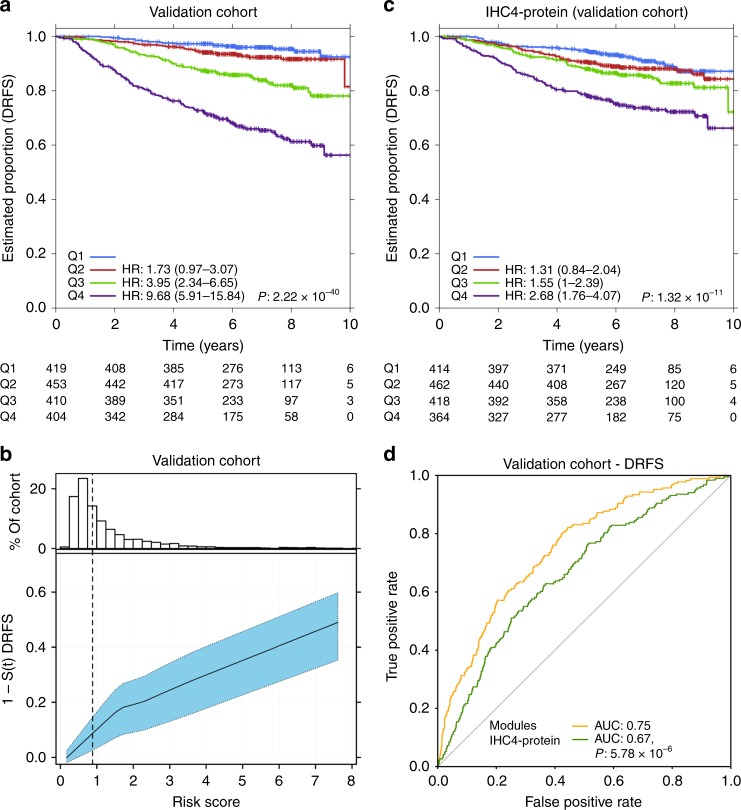
PIK3CA signaling predictor of breast cancer recurrence. **a** Independent validation of prognostic model trained on SIMMS’ risk scores and clinical covariates (N and tumor size). Risk score estimates were grouped into quartiles derived from the TEAM training cohort; each group was compared against Q1. Hazard ratios were estimated using Cox proportional hazards model and significance of survival difference was estimated using the log-rank test assessing heterogeneity across the four groups. **b** Distribution of patient risk scores in the TEAM Validation cohort (top panel). Bottom panel shows the predicted 5-year recurrence probabilities (solid line) and 95% CI (dashed lines) as a function of patient risk score. Vertical dashed black line indicates training set median risk score. **c** Risk prediction by the IHC4 protein model in the TEAM validation cohort. Quartiles were defined in the training cohort and applied to the validation cohort. Quartiles Q2-Q4 were compared against Q1, with adjustment for age, nodal status, tumor size and grade using Cox proportional hazards modeling and the log-rank test. **d** Comparison of SIMMS’ modules model (PIK3CA risk predictor) and IHC4-protein model using area under the *receiver operating characteristic* (AUC) curve as performance indicator.

To validate this model, we used a fully-independent set of 1742 patients from the same clinical trial profiled using the same technology (Supplementary Table 20). This scenario closely replicates actual clinical application of the signature. The SIMMS signature was a robust predictor of distant metastasis in the validation cohort (Fig. 5a; Q4 vs. Q1 HR = 9.68, 95%CI: 5.91–15.84; *P* = 1.71 × 10^−19^). It was also effective when simply median-dichotomizing predicted risk scores into low- and high-risk groups (Supplementary Fig. 24a). Risk scores from this signature were directly correlated with the likelihood of distant recurrence at five years, with a higher risk score associated with a higher likelihood of metastasis (Fig. 5b). The signature was independent of PIK3CA point mutations, with no change in survival curves between low and high-risk groups with vs. without PIK3CA mutations (p_low+/-_ = 0.22, p_high+/-_ = 0.81; Supplementary Fig. 24b). The signature remained an independent prognostic indicator following adjustment for chemotherapy (Q4 vs. Q1 HR = 9.88, 95%CI: 6.01–16.27; *P* = 2.02 × 10^−19^). To further verify this, predicted risk groups (Q1-Q4) in the validation cohort were divided into chemotherapy negative and positive arms with further stratification by nodal status. Risk predictions was similar for node-negative/chemotherapy-negative patients (Q4 vs. Q1 HR = 7.69, 95%CI: 3.19–18.58; *P* = 5.76 × 10^−6^; Supplementary Fig. 24c) as for node-positive/chemotherapy-negative patients (Q4 vs. Q1 HR = 8.76, 95%CI: 3.78-20.29; *P* = 4.09 × 10^−7^; Supplementary Fig. 24d), as well as for chemotherapy-stratified groups without the prior knowledge of nodal status (Supplementary Fig. 24e-f, Supplementary Methods section 4.7). This FFPE-derived risk model successfully validated in fresh-frozen ER+ clinical samples from the Metabric cohort (HR = 2.41, 95%CI: 2.01–2.89; *P* = 2.09 × 10^−21^; Supplementary Fig. 24g), despite the change in genomics platform, fixation/preservation and analyte-extraction protocols.

To benchmark SIMMS’ PI3K modules signature against current clinically-validated approaches, we compared its performance to a clinically-validated protein-based residual risk predictor, IHC4^36^. IHC4 was assessed using quantitative IHC measurements of ER, PgR, Ki67 and HER2^37^ with adjustment for age, nodal status, grade and size in both the training and validation cohorts (validation set: Wald *P* = 1.32 × 10^−11^; Fig. 5c). To compare the two predictors, we used the area under the *receiver operating characteristic* curve as a performance indicator. The PI3K modules model (AUC = 0.75) was significantly superior to the IHC-protein model (AUC = 0.67; *P* = 5.78 × 10^−6^; Fig. 5d). The PIK3CA predictor correctly identified 78.7% (NPV = 0.93, PPV = 0.27) of patients with disease relapse compared to 63.0% (NPV = 0.88, PPV = 0.22) by IHC4 in the validation cohort. Overall, it improved patient classification relative to IHC4 for 18% of patients (Net reclassification index = 0.18, 95% CI = 0.11–0.25, *P* < 2.2 × 10^−16^).

### General multi-modal biomarkers

Since oncogenic insults manifest across all molecular species (e.g., DNA, mRNA, protein), there is a need to simultaneously integrate these into unified predictive models. We used four TCGA datasets (breast (BRCA)^38^, colorectal (COADREAD)^7^, kidney (KIRC)^39^, ovarian (OV)^40^) along with the Metabric^31^ breast cancer cohort, each of which included matched mRNA, CNA and clinical data. In order to test pathways harboring multi-modal alterations, we curated previously published pathway modules (MEMo)^41^ from TCGA studies (Supplementary Table 21). SIMMS risk scores were estimated for each of the mRNA and CNA profiles with subnetwork weights of constituent genes calculated independently. The sum of mRNA and CNA MDS yielded a multi-modal pathway activation estimate per patient (Supplementary Methods section 5). Multi-modal markers of kidney (5/8) and breast (19/23) cancers were reproducibly superior (Fisher’s combined probability test) to both mRNA-alone and CNA-alone (Supplementary Fig. 25a: dark brown dots against red and blue covariates, Supplementary Fig. 25b-c). For ovarian cancer, multi-modal markers improved upon CNA models in 2/3 subnetworks (Supplementary Fig. 25a: M02 and M03 against purple covariate, Supplementary Fig. 25b-c) even though no individual data type was prognostic in all subnetworks. These results demonstrate the potential of pathway-derived subnetwork models to generate integrated multi-modal biomarkers.

## Discussion

Patients with complex human diseases present highly heterogeneous molecular profiles, ranging from a few aberrant genes to a set of dysregulated pathways. Because many different molecular aberrations can give rise to a single clinical phenotype, the importance of generating multi-modal datasets is increasingly appreciated^7,31,40,42^. Indeed, a single whole-genome sequencing experiment generates information about single nucleotide variations, copy number aberrations and genomic rearrangements. SIMMS puts this molecular variability into the context of existing knowledge of biological pathways using subnetwork information. Several other groups have considered the value of network models in predicting breast cancer outcome^10,22,30^ and in subtyping glioblastoma^43^. However no such tools have yet been developed to be generalizable to a broad range of diseases or to arbitrary topological measures that might be used to estimate weights in network-models of biology^44,45^ or to work with physical, functional, transcriptional or metabolic networks^46,47^. SIMMS provides this generalizability and flexibility by treating molecular profiles as generic features and not just genes.

Most previous biomarker studies have focused on establishing biomarkers using mRNA abundance profiles, with pathway-level analysis used *post hoc* to characterize the most interesting genes^48–50^. Our approach inverts this strategy, taking known pathways a priori and thus creating immediately interpretable and clinically actionable biomarkers^12^. We recognize that for breast and ovarian cancers, pathway-based models presented here yield similar prognostic association as for some single gene biomarkers. This phenomenon is surprising and potentially confounded to some extent by a number of factors, including differential power across disease types and inter- and intra-tumoural heterogeneity^51^. Overall, the observed saturation of prognostic signal for some disease types require further evaluation in much larger cohorts, as well as additional tumor types. Nonetheless, our data highlights that prognostic markers based on functionally related genes offer new opportunities to rationalize disease biology and foster discovery of candidate drug targets. For example, by identifying the key signaling nodes within one of the most frequently dysregulated pathways in human cancer^52^ we are able to demonstrate both improved risk stratification of patients undergoing standard of care treatments (Fig. 5) and highlight the potential for future theranostic trials for patients stratified using this approach. Both the IHC4 and type I receptor tyrosine kinase modules have extensive clinical and pre-clinical data validating their utility in early breast cancer^53,54^. The documented effects of PIK3CA pathway inhibitors in advanced breast cancer, if appropriately targeted, may be translated into significant improvements in survival in early breast cancer.

Precision molecular medicine is predicated on the concept of giving each patient the right drug in the right dose at the right time. This type of personalized treatment requires the development of robust biomarkers that precisely predict clinical phenotypes. Current clinical biomarkers are typically derived from a small number of genes, and do not yet recapitulate the full complexity of disease. SIMMS takes a step towards integrating diverse cellular processes into a singular model, and is well-positioned to take into account the influx of clinical sequencing data now being generated. However, as -omic techniques evolve to rapidly analyze and quantify cellular metabolites, network models may need to change from being gene-centric to including metabolites as core nodes. Further, single-cell analysis methods may allow accurate interrogation of the interactions between different cell-types, perhaps requiring simultaneous fitting of multiple distinct, but interacting network models. The continued development of robust, general biomarker discovery algorithms is thus required to generate the accurate and reproducible biomarkers needed for transforming medical care.

## Methods

### Pathways database pre-processing

Pathways database was downloaded from the NCI-Nature Pathway Interaction database^27^ in PID-XML format (Supplementary Table 1). The XML dataset was parsed to extract protein-protein interactions from all the pathways using custom Perl (v5.8.8) scripts (Methods: Code Availability). The protein identifiers extracted from the XML dataset were further mapped to Entrez gene identifiers using Ensembl BioMart (version 62). Wherever annotations referred to a class of proteins, all members of the class were included in the pathway, in some case using additional annotations from Reactome and Uniprot databases. The protein-protein interactions, once mapped to the Entrez gene identifiers, were grouped under respective pathways for subsequent processing. The initial dataset contained 1159 subnetworks (Supplementary Fig. 2a-b). In order to identify redundant subnetworks, we tested the overlap between all pairs of subnetworks. When a pair of subnetworks had a two-way overlap above 80% (if two modules shared over 80% of their network components; nodes and edges), we eliminated the smaller module. Additionally, all subnetworks modules containing less than 3 edges were excluded. In total, these criteria removed 659 subnetworks, resulting in 500 subnetworks.

### mRNA abundance and survival data pre-processing

All pre-processing was performed in R statistical environment (v2.13.0). Raw datasets from breast, colon, NSCLC and ovarian cancer studies (Supplementary Tables 2-5) were normalized using RMA algorithm^55^ (R package: affy v1.28.0), except for two colon cancer datasets (TCGA and Loboda dataset), which were used in their original pre-normalized and log-transformed format. ProbeSet annotation to Entrez IDs was done using custom CDFs^56^ (R packages: hgu133ahsentrezgcdf v12.1.0, hgu133bhsentrezgcdf v12.1.0, hgu133plus2hsentrezgcdf v12.1.0, hthgu133ahsentrezgcdf v12.1.0, hgu95av2hsentrezgcdf v12.1.0 for breast cancer datasets. hgu133ahsentrezgcdf v14.0.0, hgu133bhsentrezgcdf v14.0.0, hgu133plus2hsentrezgcdf v14.0.0, hthgu133ahsentrezgcdf v14.0.0, hgu95av2hsentrezgcdf v14.0.0 and hu6800hsentrezgcdf v14.0.0 for the respective colon, NSCLC and ovarian cancer datasets). The Metabric breast cancer dataset was pre-processed, summarized and quantile-normalized from the raw expression files generated by Illumina BeadStudio. (R packages: beadarray v2.4.2 and illuminaHuman v3.db_1.12.2). Raw Metabric files were downloaded from European genome-phenome archive (EGA) (Study ID: EGAS00000000083). Data files of one Metabric sample were not available at the time of our analysis, and were therefore excluded. All datasets were normalized independently. TCGA breast (BRCA), colon (COADREAD), kidney (KIRC) and ovarian (OV) cancer datasets were downloaded from http://gdac.broadinstitute.org/ (Illumina HiSeq rnaseqv2 level 3 RSEM; release 2014-01-15). The choice of training and validation sets was driven by maintaining homogeneity in size and platforms, and was further addressed through 10-fold cross validation, as well as permutation analyses. Raw mRNA abundance NanoString counts data were pre-processed using the R package NanoStringNorm^57^ (v1.1.16; Supplementary Methods section 4). A range of pre-processing schemes was assessed to optimize normalization parameters (Supplementary Methods section 4). For breast, NSCLC and ovarian cancers with different survival end-points, overall survival (OS) was used as the survival time variable; for the studies that did not report OS, we used the closest alternative endpoint available in that study (e.g., disease-specific survival or distant metastasis-free survival). For colon cancer, all studies reported relapse/disease free survival and hence this was used as the survival end-point.

### TEAM study population

The TEAM trial is a multinational, randomized, open-label, phase III trial in which postmenopausal women with hormone receptor-positive luminal^58^ early breast cancer were randomly assigned to receive exemestane (25 mg once daily), or tamoxifen (20 mg once daily) for the first 2.5-3 years followed by exemestane (total of 5 years treatment). This study complied with the Declaration of Helsinki, individual ethics committee guidelines, and the International Conference on Harmonization and Good Clinical Practice guidelines; all patients provided informed consent. Distant metastasis free survival (DRFS) was defined as time from randomization to distant relapse or death from breast cancer^58^.

The TEAM trial included a well-powered pathology research study of over 4,500 patients from five countries (Supplementary Table 15). Power analysis was performed to confirm the study size had 98.57% and 98.82% power to detect a HR of at least 2 in the training and validation cohorts, respectively, (Supplementary Methods section 4) analyses and statistical methods followed REMARK guidelines^59^. After mRNA extraction and NanoString analysis, 3476 samples were available. Patients were randomly assigned to either a training cohort (*n* = 1734) or the validation cohort (*n* = 1742) by randomly splitting the 297 NanoString nCounter cartridges into two groups. The training and validation cohorts are statistically indistinguishable from one another and from the overall trial cohort (Supplementary Table 20)^35^.

### RNA extraction

Five 4 µm formalin-fixed paraffin-embedded (FFPE) sections per case were deparaffinised, tumor areas were macro-dissected and RNA extracted according to Ambion® Recoverall™ Total Nucleic Acid Isolation Kit-RNA extraction protocol (Life Technologies^TM^, Ontario, Canada) except that samples were incubated in protease for 3 h instead of 15 minutes. RNA samples were eluted and quantified using a Nanodrop-8000 spectrophotometer (Delaware, USA). Samples, where necessary, underwent sodium-acetate/ethanol re-precipitation. We selected 33 genes of interest from key functional nodes in the PIK3CA signaling pathway^60^ and 6 reference genes (Supplementary Table 16, Supplementary Methods section 4). Probes for each gene were designed and synthesized at NanoString Technologies (Washington, USA). RNA samples (400 ng; 5 μL of 80 ng/μL) were hybridized, processed and analyzed using the NanoString nCounter® Analysis System, according to NanoString Technologies protocols.

### Meta-analysis

Following univariate analyses and elimination of redundant patients (Supplementary Methods section 1**)**, the remaining studies were divided into two sets; training and validation cohorts (Supplementary Tables 2–5). The RMA normalized mRNA abundance measures were converted to *z*-scores within the scope of each dataset (R package: stats v2.13.0).

1- Gene hazard ratio

The hazard ratio for all the genes by combining samples from all the training datasets was estimated using the univariate Cox proportional hazards model. The Cox model was fitted to the median dichotomized grouping of mRNA abundance profiles of the samples as opposed to continuous measure of mRNA abundance.

2- Interaction hazard ratio

The hazard ratio for all the protein-protein interactions gathered from the NCI-Nature pathway interaction database were estimated using a multivariate Cox proportional hazards model. A Cox model, shown below, was fitted to median dichotomized patient grouping of each of the interacting gene pairs:

1 $$h(t) =h_{0} (t) \exp (\beta_{1} X_{G1} + \beta_{2} X_{G2} + \beta_{3} X_{G1.G2})$$

where *X*_G1_ and *X*_G2_ represent patient’s risk group for gene 1 and gene 2. *X*_G1.G2_ represents patient’s binary interaction measure between the gene 1 and gene 2, as shown below:

2 $$X_{G1.G2} = (\overline {G1 \oplus G2} )$$

where ⊕ represents exclusive disjunction between the grouping of each gene. This expression encodes “XNOR” boolean function emulating *true (1)* whenever, for a given patient, both the interacting genes result in the same risk group.

### Subnetwork module-dysregulation score (MDS)

The pathway-based subnetworks modules were scored using three different models. These models estimate a module-dysregulation score (MDS) by incorporating the hazard ratio of nodes and edges that form the subnetwork, say for a given subnetwork module *k*:

1- Nodes + Edges

3 $${\mathrm{MDS}}\left( k \right) = \mathop {\sum }\limits_{i = 1}^n \left| {\log _2{\mathrm{HR}}_i} \right| + \mathop {\sum }\limits_{j = 1}^e \left| {\log _2{\mathrm{HR}}_j} \right|$$

2- Nodes only

4 $${\mathrm{MDS}}\left( k \right) = \mathop {\sum }\limits_{i = 1}^n \left| {\log _2{\mathrm{HR}}_i} \right|$$

3- Edges only

5 $${\mathrm{MDS}}\left( k \right) = \mathop {\sum }\limits_{j = 1}^e \left| {\log _2{\mathrm{HR}}_j} \right|$$

here *n* and *e* represent total number of nodes (genes) and edges (interactions) in a subnetwork, respectively. HR represents the hazard ratios of genes and the protein-protein interactions in a subnetwork (Wald *P* < 0.05) (section: Meta-analysis). The subnetworks were ranked by MDS, thereby ranking candidate prognostic features.

### Patient risk score

The subnetwork MDS was used to draw a list of the top *n* subnetwork features for each of the three models (section: Subnetwork module-dysregulation score). These features were subsequently used to estimate patient risk scores using Model N + E, N and E. For a patient (*t*), the risk score for a given subnetwork (risk_SN_) was estimated using the following models:

1 - Nodes + Edges

6 $${\mathrm{risk}}_{\left( {{\mathrm{SN}},t} \right)} = \mathop {\sum }\limits_{i = 1}^n \left( {{\mathrm{log}}_2{\mathrm{HR}}_i} \right)X_{(t,i)} + \mathop {\sum }\limits_{j = 1}^e \left( {{\mathrm{log}}_2{\mathrm{HR}}_{\mathrm{j}}} \right)X_{(t,j_x)}X_{(t,j_y)}$$

2 - Nodes only

7 $${\mathrm{risk}}_{\left( {{\mathrm{SN}},t} \right)} = \mathop {\sum }\limits_{i = 1}^n \left( {{\mathrm{log}}_2{\mathrm{HR}}_i} \right)X_{(t,i)}$$

3 - Edges only

8 $${\mathrm{risk}}_{\left( {{\mathrm{SN}},t} \right)} = \mathop {\sum }\limits_{j = 1}^e \left( {{\mathrm{log}}_2{\mathrm{HR}}_j} \right)X_{(t,j_x)}X_{(t,j_y)}$$

where *n* and *e* represent the total number of nodes (genes) and edges (interactions) in a subnetwork (SN), respectively. HR is the hazard ratio of genes and the protein-protein interactions (Wald *P* < 0.5; only to filter genes where Cox model fails to fit estimating large/unstable coefficients) (section: Meta-analysis). *x* and *y* are the two nodes connected by an edge *j* and *X* is the scaled intensity of the molecular profile being modeled (e.g., mRNA abundance, copy number aberrations, DNA methylation beta values etc) for a patient *t*.

A univariate Cox proportional hazards model was fitted to the training set, and applied to the validation set for each of the subnetworks. The prognostic ability of all three models was compared using non-parametric two sample Wilcoxon rank-sum test.

### Subnetwork feature selection

In order to prioritize an optimal combination of subnetwork features for SIMMS’ multivariate models, we fitted a Cox model using generalized linear models (*L1*-regularization) in 10-fold cross validation setting on the training cohort (R package: glmnet v1.9-8). SIMMS R package supports additional machine learning algorithms including elastic-nets (ridge to LASSO), backward elimination and forward selection (R package: MASS v7.3-12). The fitted coefficients (_*β*_) were subsequently used to estimate an overall measure of per patient risk score for the validation set using the following formula:

9 $${\mathrm{risk}}_i = \mathop {\sum }\limits_{j = 1}^m \beta _j\left( {Y_{ij}} \right)$$

where *Y*_*ij*_ is the _*i*_*th* patient’s risk score for subnetwork *j*. The training set HRs of the nodes and edges were used to estimate *Y*_*ij*_ (section: Patient risk score). Next, we median dichotomized the validation cohort into low-risk and high-risk patients (or quartiles) using the median risk score (or quartiles) estimated using the training set. The risk group classification was assessed for potential association with patient survival data using Cox proportional hazards model and Kaplan–Meier survival analysis.

### Randomization of candidate subnetwork markers

Jackknifing was performed over the subnetwork marker space for four tumor types; breast, colon, NSCLC and ovarian. Ten million prognostic classifiers (200,000 for each size *n* = 5,10,15,…., 250; where *n* represents the number of subnetworks) were randomly sampled using all 500 subnetworks. The predictive performance of each random classifier was measured as the absolute value of the log_2_-transformed hazard ratio obtained by fitting a multivariate Cox proportional hazards model using Model N.

### Code availability

Pre-processing Perl source code is freely available through zenodo 10.5281/zenodo.1303838 and SIMMS R package is freely available through CRAN: https://cran.r-project.org/web/packages/SIMMS.

### Visualizations

All plots were created in the R statistical environment (v2.13.0 or above) using R packages BPG^61^ (v5.9.2), lattice (v0.19-28), latticeExtra (v0.6-16) and VennDiagram (v1.0.0).

## Electronic supplementary material

Supplementary Information

Description of Additional Supplementary files

Supplementary Data 1

Supplementary Data 2

Supplementary Data 3

Supplementary Data 4

Supplementary Data 5

Supplementary Data 6

Supplementary Data 7

Supplementary Data 8

Supplementary Data 9

Supplementary Data 10

Supplementary Data 11

Supplementary Data 12

Supplementary Data 13

Supplementary Data 14

Supplementary Data 15

Supplementary Data 16

Supplementary Data 17

Supplementary Data 18

Supplementary data 19

Supplementary Data 19

Supplementary Data 20

Supplementary Data 21

## Data Availability

All molecular and clinical datasets described in section: “mRNA abundance and survival data pre-processing” are freely available through original publications of those studies (Supplementary Tables 2–5). Molecular and clinical data from phase III TEAM clinical trial are available from the corresponding authors upon reasonable request.
