# Supplementary material for: Pathway-based subnetworks enable cross-disease biomarker discovery
Source: Nat Commun. 2018 Nov 12;9:4746. doi: 10.1038/s41467-018-07021-3 (PMC6232113; doi:10.1038/s41467-018-07021-3)
Supplement: Supplementary file 1 — Supplementary Information [file 41467_2018_7021_MOESM1_ESM.pdf]

Pathway-Based Subnetworks Enable Cross-Disease Biomarker Discovery

Syed Haider[1,2,14], Cindy Q .Yao[1,3,4], Vicky S. Sabine[3], Michal Grzadkowski[1], Vincent Stimper[1], Maud H.W. Starmans[1,5], Jianxin Wang[1], Francis Nguyen[1,4], Nathalie C. Moon[1], Xihui Lin[1], Camilla Drake[3], Cheryl A. Crozier[3], Cassandra L. Brookes[6], Cornelis J.H. van de Velde[7], Annette Hasenburg[8], Dirk G. Kieback[9], Christos J. Markopoulos[10], Luc Y. Dirix[11], Caroline Seynaeve[12], Daniel W. Rea[6], Arek Kasprzyk[1], Philippe Lambin[5], Pietro Lio’[2], John M.S. Bartlett[3,14], Paul C. Boutros[1,4,13,14]

[1] Informatics and Biocomputing Program, Ontario Institute for Cancer Research, Toronto, M5G 0A3, Canada

[2] Computer Laboratory, University of Cambridge, Cambridge, CB3 0FD, United Kingdom

[3] Diagnostic Development Program, Ontario Institute for Cancer Research, Toronto, M5G 0A3, Canada

[4] Department of Medical Biophysics, University of Toronto, Toronto, Canada

[5] Department of Radiation Oncology (Maastro), GROW-School for Oncology and Developmental Biology, Maastricht University Medical Center, Maastricht, The Netherlands

[6] Cancer Research UK Clinical Trials Unit, University of Birmingham, Birmingham, B15 2TT, United Kingdom

[7] Leiden University Medical Center, Leiden, The Netherlands

[8] University Hospital, Freiburg, Germany

[9] Klinikum Vest Medical Center, Marl, Germany

[10] Athens University Medical School, Athens, Greece

[11] St. Augustinus Hospital, Antwerp, Belgium

[12] Erasmus Medical Center-Daniel den Hoed, Rotterdam, The Netherlands

[13] Department of Pharmacology and Toxicology, University of Toronto, Toronto, M5S 1A8, Canada

[14] Corresponding authors

Table of Contents

Supplementary Methods **4**

1. Univariate analyses reveal outliers and duplicate profiles in breast cancer **4**

2. SIMMS' comparison with other machine learning algorithms and genesets/pathway scoring methods **5**

3. SIMMS' comparison with breast, colon, NSCLC and ovarian cancer prognostic biomarkers **7**

4. SIMMS-derived PIK3CA signaling residual risk predictor in early breast cancer **8**

4.1 TEAM cohort power calculations 8

4.2 mRNA abundance data processing 8

4.3 Survival modelling 9

4.4 IHC4 model 11

4.5 Recurrence probabilities 11

4.6 Performance Assessment 11

4.7 Prognostic assessment of SIMMS PI3K modules signature in CT+/- groups 12

5. Modelling multi-modal datatypes using SIMMS **12**

6. SIMMS R package **13**

Supplementary Figure Legends **15**

Supplementary Figure 1 .... **15**

Supplementary Figure 2 .... **16**

Supplementary Figure 3 .... **18**

Supplementary Figure 4 .... **20**

Supplementary Figure 5 .... **21**

Supplementary Figure 6 .... **22**

Supplementary Figure 7 .... **24**

Supplementary Figure 8 .... **24**

Supplementary Figure 9 .... **25**

Supplementary Figure 10 .... **26**

Supplementary Figure 11 .... **27**

Supplementary Figure 12 .... **28**
Supplementary Figure 13 .... **29**
Supplementary Figure 14 .... **30**
Supplementary Figure 15 .... **31**
Supplementary Figure 16 .... **32**
Supplementary Figure 17 .... **33**
Supplementary Figure 18 .... **34**
Supplementary Figure 19 .... **35**
Supplementary Figure 20 .... **36**
Supplementary Figure 21 .... **37**
Supplementary Figure 22 .... **38**
Supplementary Figure 23 .... **39**
Supplementary Figure 24 .... **40**
Supplementary Figure 25 .... **42**
Supplementary References **44**

Supplementary Methods

1. Univariate analyses reveal outliers and duplicate profiles in breast cancer

We collated 14 mRNA abundance breast cancer datasets (**Supplementary Table 2**). Since these datasets originate from different studies and array platforms, comprehensive univariate analyses were performed to identify outlier datasets and to find patients duplicated across datasets. First, each dataset was pre-processed independently (**Methods section: mRNA abundance and survival data pre-processing**). Next, genes across all the datasets were evaluated for their prognostic ability using a univariate Cox proportional hazards model followed by the Wald-test. All the genes were subsequently ranked by the Wald-test P value within each study. The top genes across all studies were compared on multiple criterion as detailed below:

1 - Rank Product

The Rank Product[1] of each gene was computed as:

$$RP_g = \sum_{i=1}^{k} \log(r_{gi})^{\frac{1}{k}} \qquad (1)$$

Here *k* represents the number of studies which had the mRNA abundance measure available for gene *g*. r_i is the rank of gene *g* in study *i.* The overall ranking table was used as a benchmark to identify datasets in which a given gene was ranked farthest when its rank product was compared to studywise ranks. The farthest dataset count was computed for the overall top ranked (100, 200, 300,…, 1000, 2000) genes (**Supplementary Figure 3a-e**).

2 - Percentile ranks

The P value (Wald-test) based ranking was transformed into percentile ranks within each study. These ranks were used as a measure of gene's position with reference to the benchmark rank derived in the step 1 to evaluate deviation of genes' ranks for each study (**Supplementary Figure 3f-i**).

3 - Intra- and inter-study correlation

The mRNA abundance profiles of common genes across all studies were extracted and patient wise Spearman rank correlation coefficient was estimated. The correlation coefficient was used to further analyze intra- and inter-study correlation in order to identify any outlier studies (**Supplementary Figure 3j-l**).

Using the above three assessment mechanisms datasets Li and Loi were excluded. We also used correlation between individual mRNA abundance profiles in order to identify potentially redundant patients across studies. This caters for patients which might have participated in more than one study or duplicate data used in multiple studies. The survival data of patients with extremely high correlation coefficient (Spearman's $\rho \geq 0.98$) was matched, and we found 22 samples[2, 3] having identical survival time and status. These patients were removed from further analyses (**Supplementary Figure 3m**).

Cohorts of primary colon, lung and ovarian cancer patient mRNA profiles were assembled in similar ways, however, without outlier detection due to relatively small number of publicly available datasets and no (data curation based) evidence of sample sharing between studies (**Supplementary Tables 3-5**).

2. SIMMS' comparison with other machine learning algorithms and genesets/pathway scoring methods

In order to benchmark the prognostic ability of subnetworks by SIMMS' model N; using genes in each subnetwork, we fitted a Cox proportional hazard model using forward selection, backward elimination (R package: MASS v7.3-47), LASSO *L1* regularization, and ridge regularization (R package: glmnet v2.0-10), as well as a random survival forest (R package: randomForestSRC v2.5.0). To tune the hyperparameter of the regularized Cox models and the random survival forest we applied a grid search algorithm based on cross-validation in the training sets. The final models were tested in the validation cohorts and predicted risk scores of the Cox models (and the average cumulative hazards of the random survival forest) were tested for association with patient outcome. The resulting

hazard ratios and respective P values (Wald test) are analog to those presented in **Supplementary Tables 6b,7b,8b,9b** obtained by SIMMS.

To compare SIMMS prognostic performance against other genesets/pathway summary scoring methods, we chose four methods representing three different classes of scoring (CORGs: *t-test* based feature selection yielding pathway activation scores[4], Guo: geneset's summary scores[5] and PCA score: geneset's principal component scores[6]). Briefly, CORGs' pathway activation scores with embedded t-statistic based feature selection were estimated using training datasets for each cancer type. Using selected features from training set, pathway activation scores were estimated for validation datasets. For CORGs, good and poor outcome samples were determined using survival time cut-off of: breast = 5 years, colon = 5 years, NSCLC = 3 years and ovarian = 3 years removing any samples censored prior to the cut-off time; consistent with other analyses in this manuscript. Guo et al. scores were estimated using mean and median expression levels of genes in a given subnetwork, yielding effectively two different scores which were treated as two independent methods. PCA scores were estimated by using the first principal component as representative summary measure of genes in a subnetwork, which is analogous to estimates used in Bild et al.[6] and gsdScore[7].

For each subnetwork scoring method, Cox proportional hazards model was fitted on training datasets and applied to predict risk scores using validation datasets. These predicted risk scores were dichotomised on training set median risk score and resulting groups were tested for association with patient outcome using Cox model. The results of Cox model were compared across various methods and SIMMS Model N.

For sensitivity, 'positive' subnetworks were defined as those having at least three genes significantly associated with patient outcome in the training datasets (Wald-test $P < 0.05$). Here, mRNA abundance of each gene was dichotomised into low- and high-risk groups and tested for survival association using a univariate Cox proportional hazards model. Using validation datasets, the

proportion of correctly recovered subnetworks (Wald-test $P < 0.05$) by each method were regarded as true positive rate.

3. SIMMS' comparison with breast, colon, NSCLC and ovarian cancer prognostic biomarkers

In order to compare the performance of SIMMS' with existing gene expression-based breast[8], colon[9, 10], NSCLC[11-15] and ovarian[16-19] cancer prognostic biomarkers, we limited our search to the studies which shared the validation datasets with those included in our analysis as validation datasets. This selection criterion enabled unbiased comparison of hazard ratios and P values between published markers and those identified by SIMMS for the same cohorts unless specified otherwise. To maintain parity, strictly gene expression-based predictors estimating hazard ratios were included for comparison with SIMMS. These results are presented in **Supplementary Table 14**. For breast cancer biomarker, previously published[8] assessment of 9 breast cancer risk predictors were compared against the same set of ER+ breast cancer patients in Metabric Training cohort (n=801). For consistent comparison, SIMMS classifier was trained on Metabric Validation cohort, and validated on Metabric Training cohort predicting exactly the same (5-year) overall survival end-point as used by the Zhao et al[8]. To test the colon cancer 34-gene signature[10] on TCGA cohort, this signature was re-implemented following the original protocol. Briefly, VMC and Moffitt sub-cohorts were treated as training and validation sets respectively. The validation results on the Moffitt cohort (Smith) and TCGA cohort are recorded in **Supplementary Table 14**. NSCLC validation was limited to lung adenocarcinomas only. Both NSCLC and ovarian cancer comparisons were performed in the similar way maintaining the validation cohorts for coherent comparison. TCGA RNA-Seq data was used as colon and ovarian cancers validation cohorts, however, panel of other markers used microarray-based profiles for these two cohorts.

SIMMS identified markers of ER+ breast cancer compared favourably to nine other breast cancer markers of clinical outcome (**Supplementary Table 14**).

SIMMS produced the best prognostic marker for colon cancer by a wide margin compared to two other markers of patient outcome (**Supplementary Table 14**). Similar trend of enhanced performance was observed for NSCLC (adenocarcinomas) markers where SIMMS outperformed seven other markers in 3/4 independent validation studies (**Supplementary Table 14**).

4. SIMMS-derived PIK3CA signaling residual risk predictor in early breast cancer

4.1 TEAM cohort power calculations

Power calculations were performed on complete TEAM cohort (n = 3,476; events = 507) and for each of the training (n = 1,734; events = 250) and validation (n = 1,742; events = 257) subsets separately. Power estimates representing the likelihood of observing a specific HR against the above-mentioned events, (assuming equal-sized patient groups) were derived using the following formula (2):

$$z_{power} = \frac{\sqrt{E} \times \ln(HR)}{2} - z(1-\frac{\alpha}{2}) \qquad (2)$$

where *E* represents the total number of events (DRFS) and α represents the significance level which was set to 10^{-3}. z_{power} was calculated for HR ranging from 1 to 3 with steps of 0.01.

4.2 mRNA abundance data processing

Raw mRNA abundance counts data were pre-processed using R package NanoStringNorm[20] (v1.1.16). In total, 252 pre-processing schemes were evaluated; parameterising normalization with respect to six positive controls, eight negative controls and six housekeeping genes (GUSB, PUM1, SF3A1, TBP, TFRC and TMED10) followed by global normalization. To identify the

optimal pre-processing parameters, two criteria were defined. First, each of the 252 pre-processing schemes was ranked based on their ability to maximize Euclidean distance of ERBB2 mRNA abundance between HER2-positive and HER2-negative samples. The process was repeated for 1000 random subsets of HER2-positive and HER2-negative samples for each of the pre-processing schemes. Second, using 37 replicates of an RNA pool extracted from 5 randomly selected anonymized FFPE breast tumour samples, pre-processing schemes were ranked based on inter-batch variation. To this end, mixed effects linear models were used and residual estimates were used as a measure of inter-batch variation (R package: nlme v3.1-113). Cumulative ranks based on these two criteria were estimated using RankProduct[1] resulting in selection of an optimal pre-processing scheme of normalisation to the *geometric mean* derived from all genes followed by *rank normalisation.* Samples with RNA content |z-score| > 6 were discarded as being potential outliers. Only one sample was removed from the top pre-processing scheme. Six samples were run in duplicates, and their raw counts were averaged and subsequently treated as a single sample. Training and validation cohorts were created by randomly splitting 297 NanoString nCounter cartridges into two groups (**Supplementary Table 20**), which ensures that there are no batch-effects shared between the two cohorts.

4.3 Survival modelling

Univariate survival analysis of mRNA abundance profiles was performed by median-dichotomizing every gene's mRNA abundance into high- and low-abundance groups (**Supplementary Table 16**), except for *ERBB2* where risk

groups were determined via expectation-maximization clustering (*k*=2) because of the presence of a well-established sub-population of *ERBB2* expressing cancers (<15%) which are regarded as HER2/ERBB2 positive tumours. Survival analysis of clinical variables modelled age as a binary variable (dichotomized at age ≥55 as a surrogate for menopausal status), while grade, nodal status and tumour size were modelled as ordinal variables (**Supplementary Table 17**). For mRNA and IHC4 models, tumour size was treated as a continuous variable. Univariate survival analysis of mutational profiles (*AKT1*, *PIK3CA* and *RAS*;[21]) was performed by dichotomizing patients into mutant and wild-type groups.

Risk score profiles (**Methods**) of patients in the Training cohort were used to fit a multivariate Cox proportional hazards model alongside clinical variables. Given the small number of variables to select from (continuous = 9, factors = 3) and a mix of continuous and ordinal variables, we chose backwards step-wise refinement algorithm (AIC penalty term: *k* = 1 degrees of freedom) and created a module-based risk model (**Supplementary Table 19**). The parameters estimated by the multivariate model (Training cohort) were applied to the patients in the Validation cohort generating per-patient risk score. These risk scores (continuous) were grouped into quartiles using the thresholds derived from the Training cohort, and resulting groups were subsequently evaluated through Kaplan-Meier analysis. All models were trained and validated using DRFS truncated to 10 years as an end-point. All survival modelling was performed in the R statistical environment (R package: survival v2.37-4).

4.4 IHC4 model

IHC4-protein risk scores were calculated as described by Cuzick *et al.*[22], and then adjusted for clinical covariates (age, nodal status, grade and tumour size). Model predictions (continuous risk scores) were separated into quartiles (**Figure 5c**) and analysed using Kaplan-Meier analysis and multivariate Cox proportional hazards model adjusted for clinical variables.

4.5 Recurrence probabilities

Recurrence probabilities at 5 years were estimated by binning the predicted risk scores in 25 equal groups. For each group, recurrence probability $R_{(t)}$ was estimated as $1-S_{(t)}$, where $S_{(t)}$ is the Kaplan-Meier survival estimate at year 5. The $R_{(t)}$ estimates of 25 groups were smoothed using local polynomial regression fit. The predicted estimates were plotted against the median risk score of each group except the first and last group, where the lowest risk score and 99th percentile were used, respectively.

4.6 Performance Assessment

Performance of survival models was compared through area under the *receiver operating characteristic* (ROC) curve. Significance of difference between the ROC curves was assessed through permutation analysis (10,000 permutations by shuffling the risk scores while maintaining the order of survival objects). Patients censored before 5 years (Training cohort: n = 192, Validation cohort: n = 181) were eliminated from sampling. For percentage concordance analysis, patients with a relapse (after removing the afore-mentioned patients) were considered as high risk and the rest of the patients were classed as low risk

patients. Median dichotomised risk groups determined by PIK3CA predictor and IHC4 predictor were compared against the high and low risk patients. The percentage of predictions matching the high and low risk groups were regarded as concordant. ROC analysis was implemented using R packages pROC (v1.6.0.1) and survivalROC (v1.0.3). Using the same median dichotomised risk groups and actual high and low risk groups, Net reclassification improvement for PIK3CA predictor over IHC4 predictor was estimated using the R package PredictABEL (v1.2-1).

4.7 Prognostic assessment of SIMMS PI3K modules signature in CT+/- groups

SIMMS-derived PI3K modules signature was evaluated in chemotherapy-stratified groups without the prior knowledge of nodal status. Patients in the highest risk quartile (Q4) showed significantly decreased survival rate compared to low risk patients, independent of whether they received chemotherapy (Q4 *vs.* Q1 HR=11.07, 95%CI: 3.47-35.26; $P=5.29 \times 10^{-11}$) (**Supplementary Figure 24e**) or did not (Q4 *vs.* Q1 HR=9.74 95%CI: 5.58-17.02; $P=1.66 \times 10^{-29}$) (**Supplementary Figure 24f**).

5. Modelling multi-modal datatypes using SIMMS

Recent studies conducted by TCGA have generated datasets on matched genomic and transcriptomic profiles including mutations, copy-number aberration (CNA), DNA methylation and mRNA abundance[17, 23]. These datasets can potentially lead to the discovery of new biomarkers bridging the gap between multi-modal molecular features and clinical covariates. To test this, we curated previously published pathway modules (MEMo[24]) from TCGA studies harbouring multiple aberrations (e.g. somatic mutations, somatic copy-number aberrations, dysreulated mRNA abundance levels, and DNA methylation levels)[17, 25-27]. The

combined database was composed of 23 breast, 1 colorectal, 8 kidney renal clear cell and 3 ovarian cancer modules (**Supplementary Table 21**). Using these modules, SIMMS' (Model N) breast cancer risk predictors were created using 1000 randomly generated subsets (50% samples) of Metabric cohort and validated on the held-out Metabric subsets as well as TCGA breast cancer cohort. Similarly, 1000 randomly generated subsets (50% samples) of TCGA colorectal, kidney renal clear cell carcinoma and ovarian cancers were used to train and validate (50% held-out samples) the prognostic ability of each of the subnetwork modules. The results of 1000 models per cancer type were summarised using Fisher's method (Fisher's combined probability test) resulting in a chi-square estimate and a P value. We used molecular features based on mRNA and CNA as gene-level properties. Copy number levels -2 (homozygous deletion) and -1 (heterozygous deletion) were collapsed into one group, whilst gene copy-number levels 1 (gain) and 2 (amplification) were collapsed into a single group. Copy-number levels were modelled using Cox proportional hazards model and compared against the baseline copy number of 0 (diploid). Additional filter of minimum 3% copy-number aberration frequency in the cohort in at least one group (gain/amplification and deletion) was applied prior to estimating parameters for each gene, failing which would mean gene's copy-number changes would not contribute to SIMMS' risk scores. Overall survival was used as survival end-point for all cancer types analysed in the multi-modal modelling.

6. SIMMS R package

SIMMS is implemented in R and is available under the GNU General Public License (GPL) version 2 through CRAN: https://cran.r-project.org/web/packages/SIMMS. SIMMS is generic and can work with any combination of molecular features and interaction networks. It provides an extendible framework to support user-defined parameter estimation and classification algorithms. The R package of SIMMS offers three key features: (i) support for multiple datatypes (mRNA, methylation, CNA etc), (ii) support for user-defined networks, and (iii) support for user-defined methods for quantifying dysregulation of a subnetwork. For (i), users can supply the location and names

of the files they would like to analyze with SIMMS. For (ii), a text file describing networks in a tab-delimited format can be supplied as an input to SIMMS, see pathway_based_networks*.txt files that comes as a part of R package. For (iii), the package offers an interface function 'derive.network.features' that accepts a parameter 'feature.selection.fun' for user-defined function name (see code snippet below). By default, the function 'calculate.network.coefficients' is called to estimate MDS and risk scores for Mode N, Model E and Mode N+E as described in this paper. However, users can easily write their own algorithms and simply use them with SIMMS as a plug and play component. For details, see package manual and vignettes.

```
derive.network.features <- function(
     data.directory = ".",
     output.directory = ".",
     data.types = c("mRNA"),
     feature.selection.fun = "calculate.network.coefficients",
     feature.selection.datasets = NULL,
     feature.selection.p.thresholds = c(0.05),
     subset = NULL, ...
     );
```

Supplementary Figure Legends

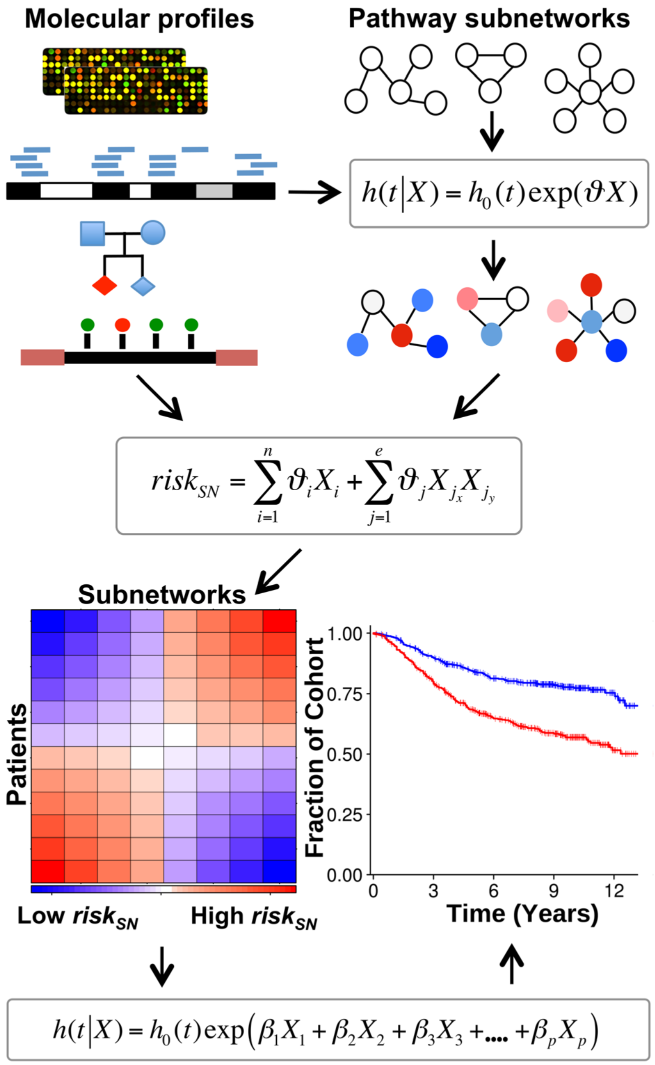

Supplementary Figure 1

Schematic overview of SIMMS. Subnetwork modules were extracted from NCI-Nature/Biocarta/Reactome curated pathways by isolating protein-protein interaction networks within a pathway. Molecular profiles were systemised and split into independent training and validation sets. Each extracted subnetwork was scored (module-dysregulation score) using 3 different models and ranked. High-ranking subnetworks were used to compute a patient-wise risk score. Most optimal combination of predictive subnetworks was selected using a machine

learning algorithm with built-in options of generalized linear models with elastic-nets parameter alpha (α) supporting ridge to LASSO *L1*-regularization ($\alpha \in [0,1]$), Backward elimination and Forward selection algorithms, resulting in a multivariate subnetwork-based classifier. The classifier is then tested on independent validation sets.

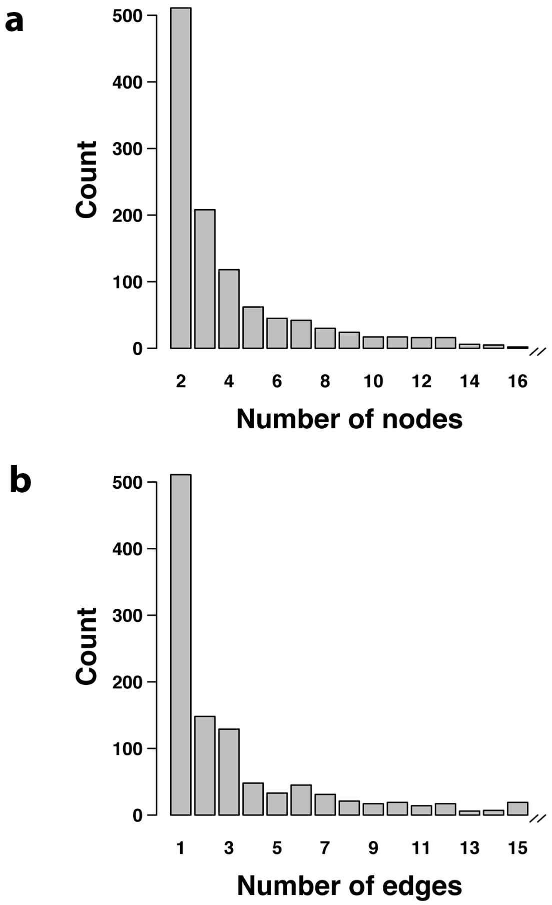

Supplementary Figure 2

Summary of pathways database. Distribution of nodes **(a)** and edges **(b)** across all subnetwork modules extracted from NCI-Nature curated pathways (Reactome and Biocarta inclusive).

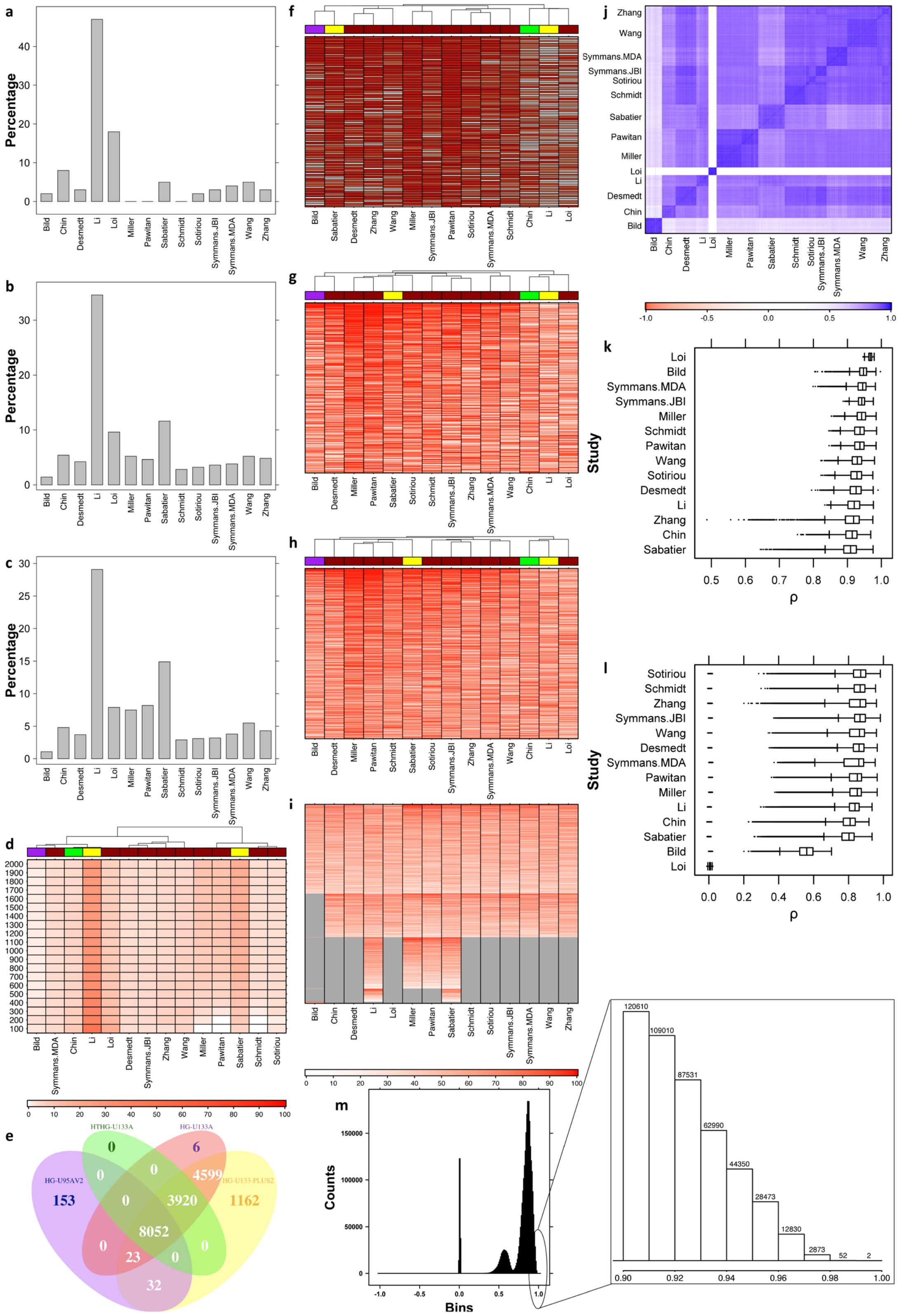
a
b
c
d
e
f
g
h
i
j
k
l
m
Percentage
Bild
Chin
Desmedt
Li
Loi
Miller
Pawitan
Sabatier
Schmidt
Sotiriou
Symmans.JBI
Symmans.MDA
Wang
Zhang
Study
ρ
Counts
Bins
HG-U95AV2
HTHG-U133A
HG-U133A
HG-U133-PLUS2
153
0
6
1162
4599
3920
8052
23
32
120610
109010
87531
62990
44350
28473
12830
2873
52
2

Supplementary Figure 3

Quality assessment and identification of repeated patient profiles. (a,b,c) A univariate Cox model was fit to each gene in each study in the breast cancer cohort. Genes were ranked according to their P value (Wald-test), and a cumulative rank for all the genes was estimated using the *rank product* for each gene. The top ranked 100 (a), 500 (b) and 1,000 (c) genes were used to identify the study in which each gene was farthest away from the cumulative rank. The frequency of a study being farthest was recorded for each of the top ranked 100, 500 and 1,000 genes. Li and Loi datasets seem to be notable outliers. As the threshold is relaxed, Sabatier dataset also begins to show deviation compared to other datasets.

(d) Heatmap showing a summary of barplots (a-c) of the top ranked (rank product) 100 to 2000 genes with the percentage measure as the frequency of each dataset being the farthest from the rank product of top *n* genes. The covariates represent different microarray platforms: HG-U95AV2=purple, HTHG-U133A=green, HG-U133A=red, HG-U133-PLUS2=yellow.

(e) 4-way Venn diagram representing overlap of genes across the four Affymetrix array platforms used in the 14 breast cancer datasets included in this study. Note that the Bild dataset (array platform: HG-U95AV2) has the least number of genes (8,260) with 8,052 genes that exist across all array platforms. The analysis in a-d was done on this common gene set only.

(f,g,h) Gene ranks transformed into percentile ranks within all studies. The rank product based top 100 (f), 500 (g), and 1,000 (h) genes shown in terms of their percentile rank within each study. Li, Loi and Chin datasets clustered together and had lower percentile ranks compared to other datasets. However, Sabatier's percentile ranks were similar to other datasets thereby deemed suitable for inclusion in this study.

(i) Summary heatmap of percentile ranks across all studies, ordered by groups of genes common across studies, thereby maintaining coherent comparison of ranks.

(j) Heatmap of Spearman correlation between patients' mRNA abundance profiles. Loi dataset quite clearly shows weak correlation with the other datasets, again reflecting unusual expression patterns compared to other datasets.

(k,l) Box-whisker plots of intra- (k) and inter-study (l) correlation between patients' mRNA abundance profiles. The results show distinctively strong correlation within Loi dataset (k) and weak correlation between Loi and other datasets (l). Boxplot lines show lower quartile, median and upper quartile. Whiskers extend to the point closest to the upper/lower quartile $\pm$ (1.5 x IQR).

(m) Histogram of Spearman correlation of patients' mRNA abundance profiles. From left to right, the first peak represents correlation between Loi and other datasets. The second peak represents correlation between Bild and other datasets, while the third peak constitutes the correlation between the remaining datasets. The survival data of highly correlated profiles (zoomed in panel, $0.98 \leq \rho \leq 1.00$) was further inspected, resulting in 22 patients that were found in both Sotiriou and Symmans (JBI) datasets having identical survival data. These were removed from Symmans (JBI) dataset for further analysis.

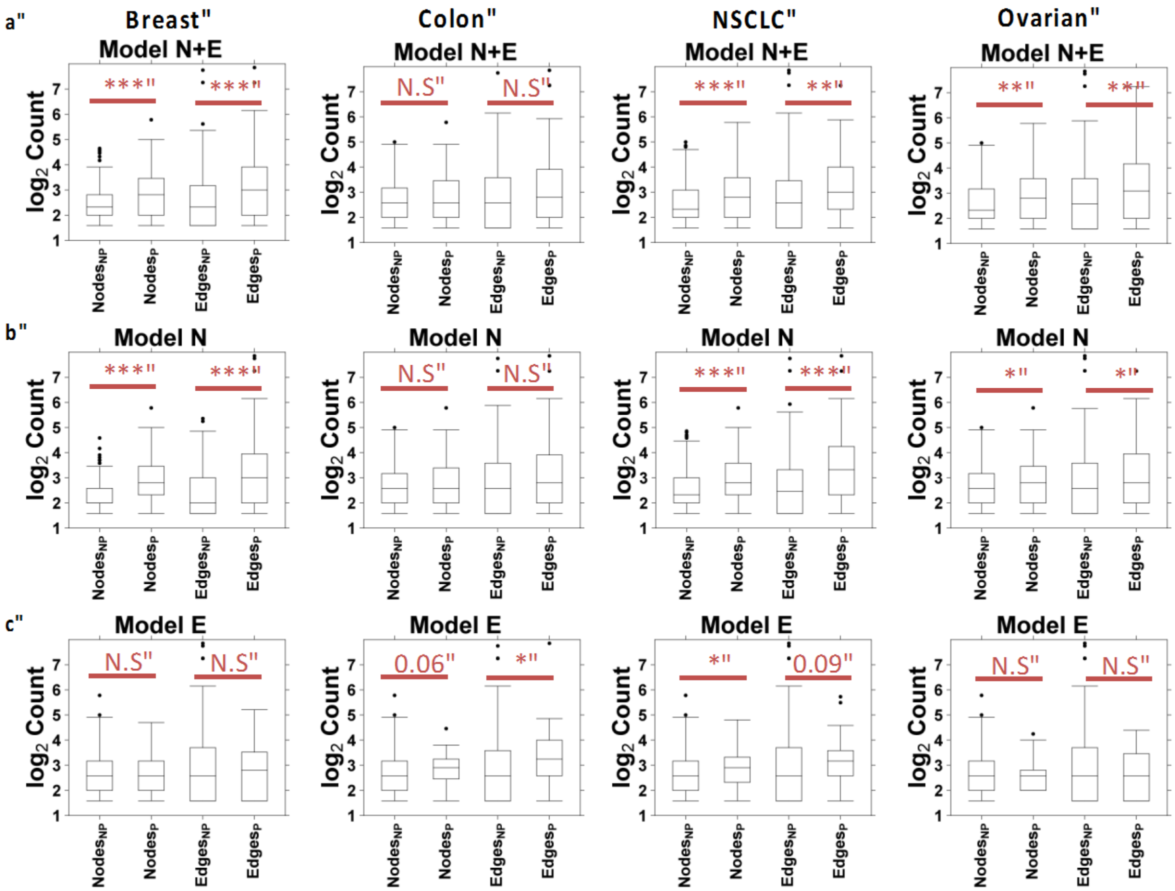

Supplementary Figure 4

Distribution of prognostic ability versus the size of subnetworks. (a-c) For each of the three scoring schemes *i.e.* Model N+E, Model N and Model E (see Methods), distribution of subnetwork size for prognostic **(*P*)** (Wald test P < 0.05; validation cohorts) and not prognostic (***NP***) subnetwork modules. Size of a subnetwork was defined in terms of number of nodes and number of edges. Pairwise comparisons were performed using Wilcox rank sum test (* P<0.05, ** P<0.01, *** P<0.001, N.S P>0.1). Boxplot lines show lower quartile, median and upper quartile. Whiskers extend to the point closest to the upper/lower quartile ± (1.5 x IQR).

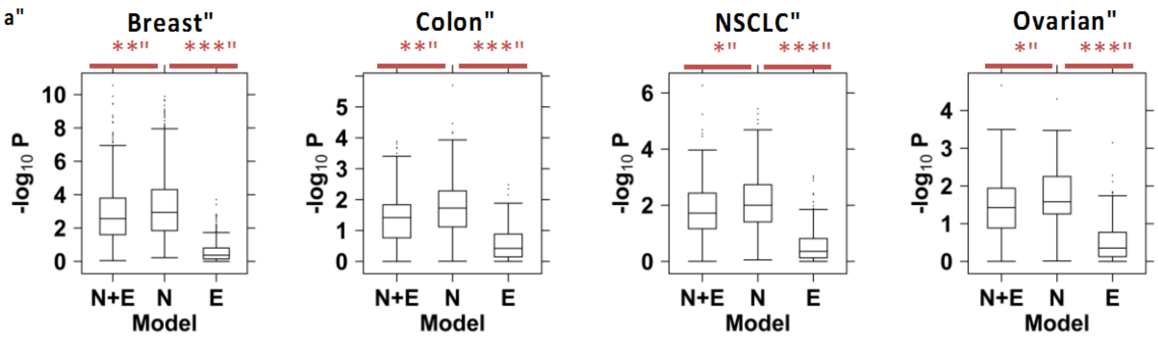

Supplementary Figure 5

Prognostic ability of SIMMS' models (a) Distribution of prognostic ability (-$\log_{10}$P) of subnetwork modules which were significant (Wald test P < 0.05) in at least one scoring scheme (Model N+E, Model N and Model E), in respective cancer type. -$\log_{10}$P values were compared using one-way ANOVA (P < 0.05) followed by Tukey HSD test. Tukey HSD test's adjusted P values for only Model N vs Model N+E and Model E are displayed (* P<0.05, ** P<0.01, *** P<0.001). Boxplot lines show lower quartile, median and upper quartile. Whiskers extend to the point closest to the upper/lower quartile ± (1.5 x IQR).

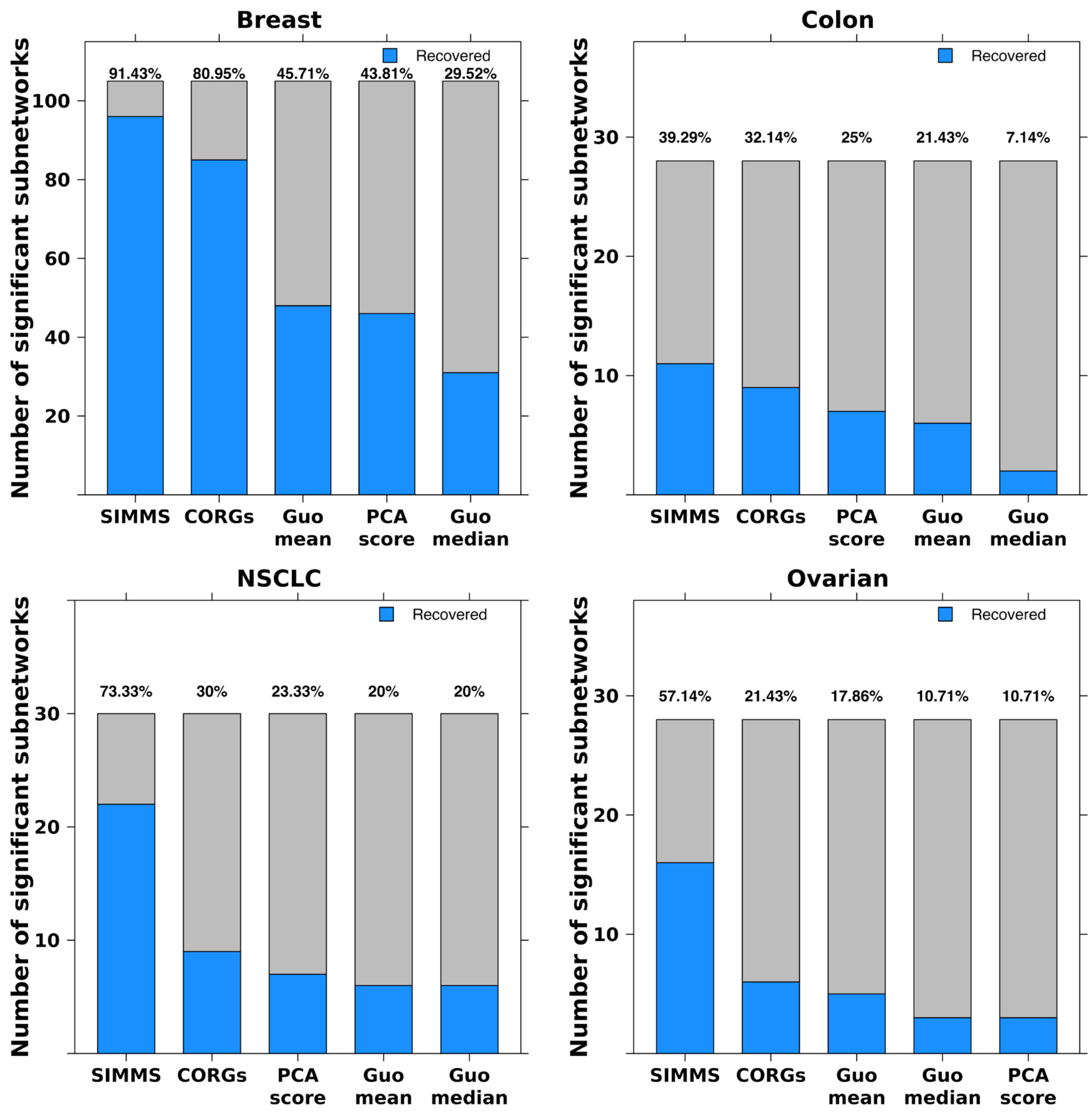

Supplementary Figure 6

Comparison of subnetwork scoring methods. Sensitivity assessment of correctly recovered ‘positive’ subnetwork modules (those likely to be associated with patient outcome) by various subnetwork/pathway scoring methods. Height of each bar represents total number of ‘positive’ subnetworks, while the blue colour shows proportion of correctly recovered ‘positive’ subnetworks. Numbers above the bars represent % true positive rate.

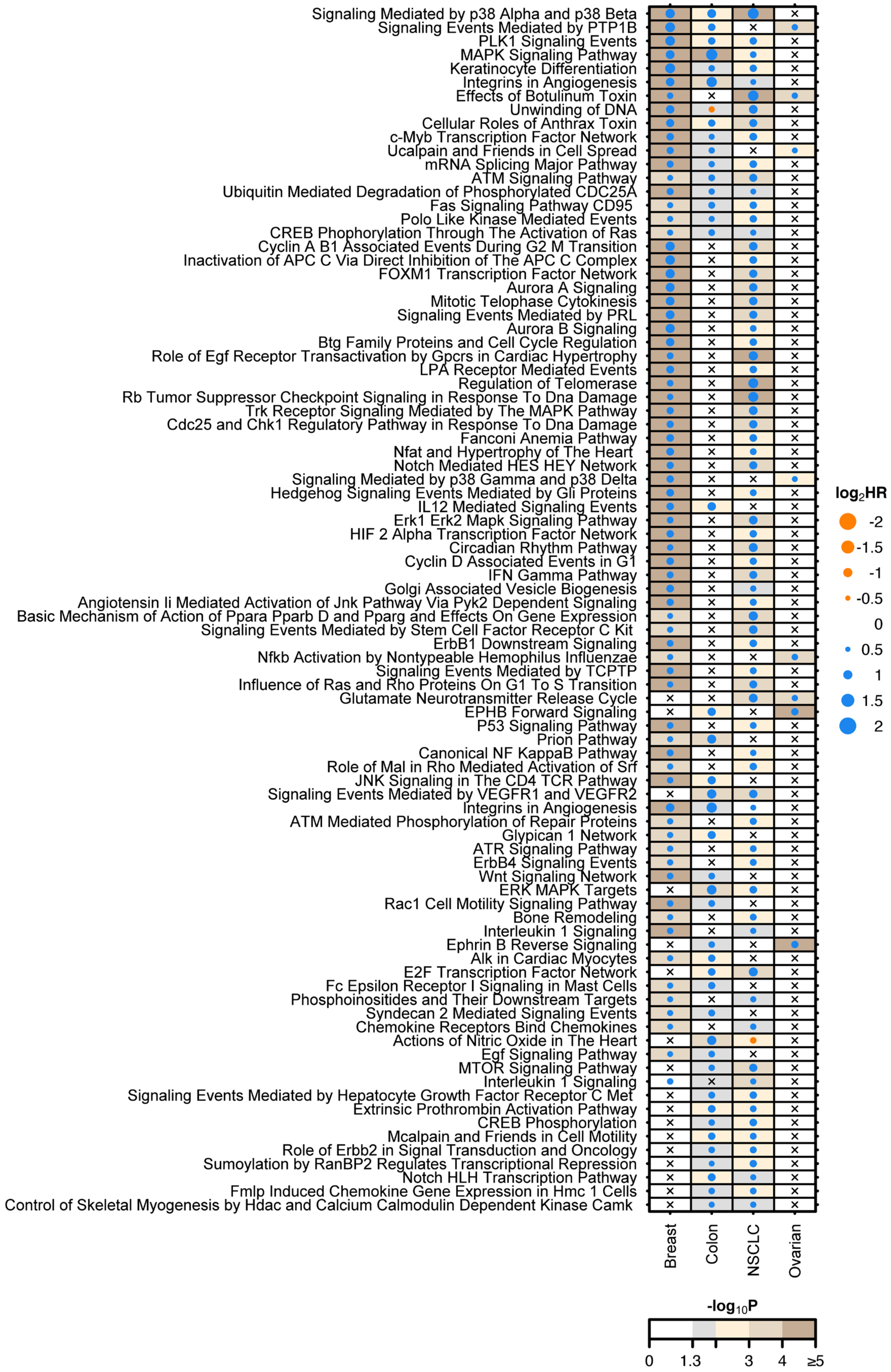
Signaling Mediated by p38 Alpha and p38 Beta
Signaling Events Mediated by PTP1B
PLK1 Signaling Events
MAPK Signaling Pathway
Keratinocyte Differentiation
Integrins in Angiogenesis
Effects of Botulinum Toxin
Unwinding of DNA
Cellular Roles of Anthrax Toxin
c-Myb Transcription Factor Network
Ucalpain and Friends in Cell Spread
mRNA Splicing Major Pathway
ATM Signaling Pathway
Ubiquitin Mediated Degradation of Phosphorylated CDC25A
Fas Signaling Pathway CD95
Polo Like Kinase Mediated Events
CREB Phophorylation Through The Activation of Ras
Cyclin A B1 Associated Events During G2 M Transition
Inactivation of APC C Via Direct Inhibition of The APC C Complex
FOXM1 Transcription Factor Network
Aurora A Signaling
Mitotic Telophase Cytokinesis
Signaling Events Mediated by PRL
Aurora B Signaling
Btg Family Proteins and Cell Cycle Regulation
Role of Egf Receptor Transactivation by Gpcrs in Cardiac Hypertrophy
LPA Receptor Mediated Events
Regulation of Telomerase
Rb Tumor Suppressor Checkpoint Signaling in Response To Dna Damage
Trk Receptor Signaling Mediated by The MAPK Pathway
Cdc25 and Chk1 Regulatory Pathway in Response To Dna Damage
Fanconi Anemia Pathway
Nfat and Hypertrophy of The Heart
Notch Mediated HES HEY Network
Signaling Mediated by p38 Gamma and p38 Delta
Hedgehog Signaling Events Mediated by Gli Proteins
IL12 Mediated Signaling Events
Erk1 Erk2 Mapk Signaling Pathway
HIF 2 Alpha Transcription Factor Network
Circadian Rhythm Pathway
Cyclin D Associated Events in G1
IFN Gamma Pathway
Golgi Associated Vesicle Biogenesis
Angiotensin Ii Mediated Activation of Jnk Pathway Via Pyk2 Dependent Signaling
Basic Mechanism of Action of Ppara Pparb D and Pparg and Effects On Gene Expression
Signaling Events Mediated by Stem Cell Factor Receptor C Kit
ErbB1 Downstream Signaling
Nfkb Activation by Nontypeable Hemophilus Influenzae
Signaling Events Mediated by TCPTP
Influence of Ras and Rho Proteins On G1 To S Transition
Glutamate Neurotransmitter Release Cycle
EPHB Forward Signaling
P53 Signaling Pathway
Prion Pathway
Canonical NF KappaB Pathway
Role of Mal in Rho Mediated Activation of Srf
JNK Signaling in The CD4 TCR Pathway
Signaling Events Mediated by VEGFR1 and VEGFR2
Integrins in Angiogenesis
ATM Mediated Phosphorylation of Repair Proteins
Glypican 1 Network
ATR Signaling Pathway
ErbB4 Signaling Events
Wnt Signaling Network
ERK MAPK Targets
Rac1 Cell Motility Signaling Pathway
Bone Remodeling
Interleukin 1 Signaling
Ephrin B Reverse Signaling
Alk in Cardiac Myocytes
E2F Transcription Factor Network
Fc Epsilon Receptor I Signaling in Mast Cells
Phosphoinositides and Their Downstream Targets
Syndecan 2 Mediated Signaling Events
Chemokine Receptors Bind Chemokines
Actions of Nitric Oxide in The Heart
Egf Signaling Pathway
MTOR Signaling Pathway
Interleukin 1 Signaling
Signaling Events Mediated by Hepatocyte Growth Factor Receptor C Met
Extrinsic Prothrombin Activation Pathway
CREB Phosphorylation
Mcalpain and Friends in Cell Motility
Role of Erbb2 in Signal Transduction and Oncology
Sumoylation by RanBP2 Regulates Transcriptional Repression
Notch HLH Transcription Pathway
Fmlp Induced Chemokine Gene Expression in Hmc 1 Cells
Control of Skeletal Myogenesis by Hdac and Calcium Calmodulin Dependent Kinase Camk
Breast
Colon
NSCLC
Ovarian
log2HR
-2
-1.5
-1
-0.5
0
0.5
1
1.5
2
-log10P
0
1.3
3
4
≥5

Supplementary Figure 7

Prognostic assessment of SIMMS' predicted risk scores. Dot plot of hazard ratios and P values of subnetwork modules significant in at least 2/4 cancer types. A Cox proportional hazards model was fitted to dichotomous risk scores (threshold derived from the training cohort) across the entire validation cohort. Crosses represent absence of subnetwork module from a particular cancer type.

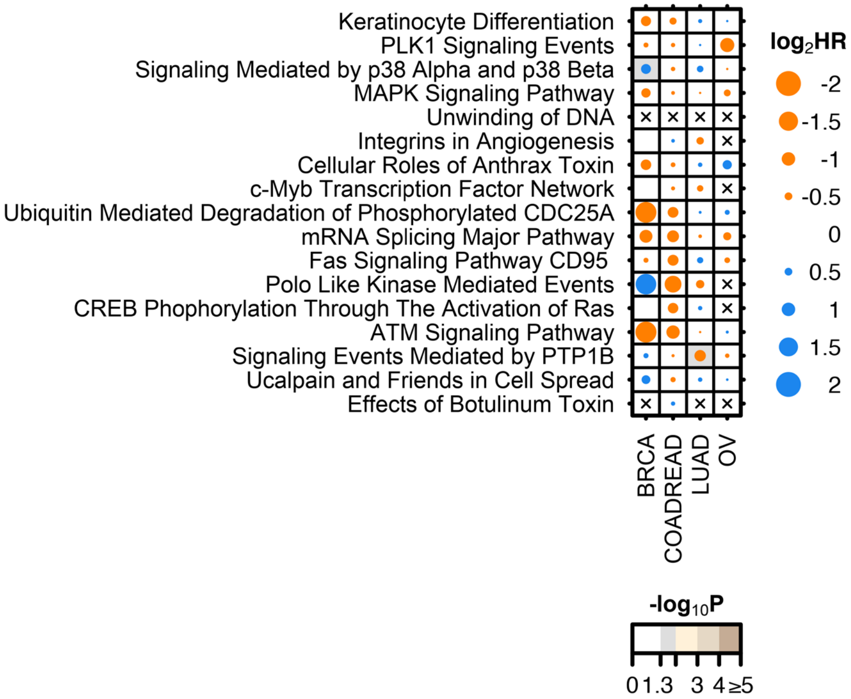

Supplementary Figure 8

Prognostic assessment of mutation burden. Dot plot of hazard ratios and P values of subnetwork modules in **Figure 1i**. Using TCGA datasets for breast, colorectal, lung adenocarcinoma and ovarian cancers; for each of these subnetwork modules (using mutations in genes involved), patients were assigned to mutant group if any gene in the subnetwork was mutated, otherwise to non-mutant group. A Cox proportional hazards model was fitted to test association of these groups with patient outcome.

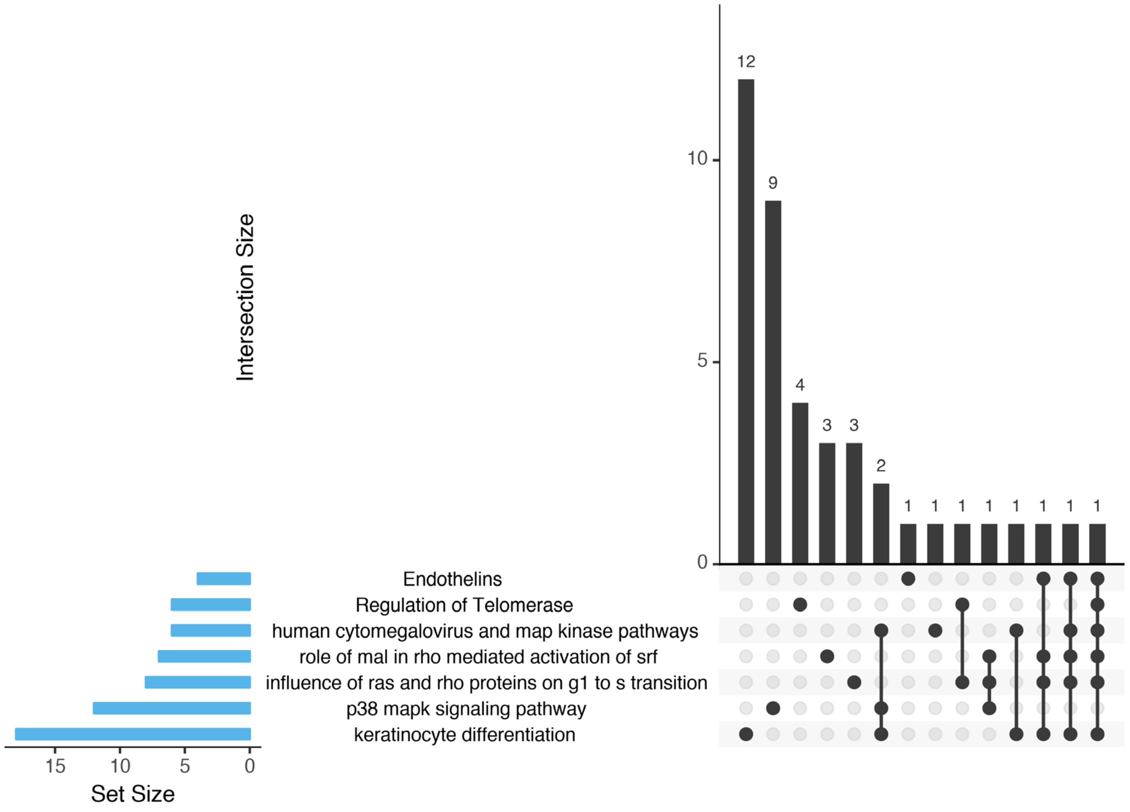

Supplementary Figure 9

Overlap of genes in subnetworks with both prognostic and predictive ability. Upset plot showing overlap of genes between subnetworks which showed significant prognostic as well as predictive (platinum response) association in TCGA ovarian cancer cohort.

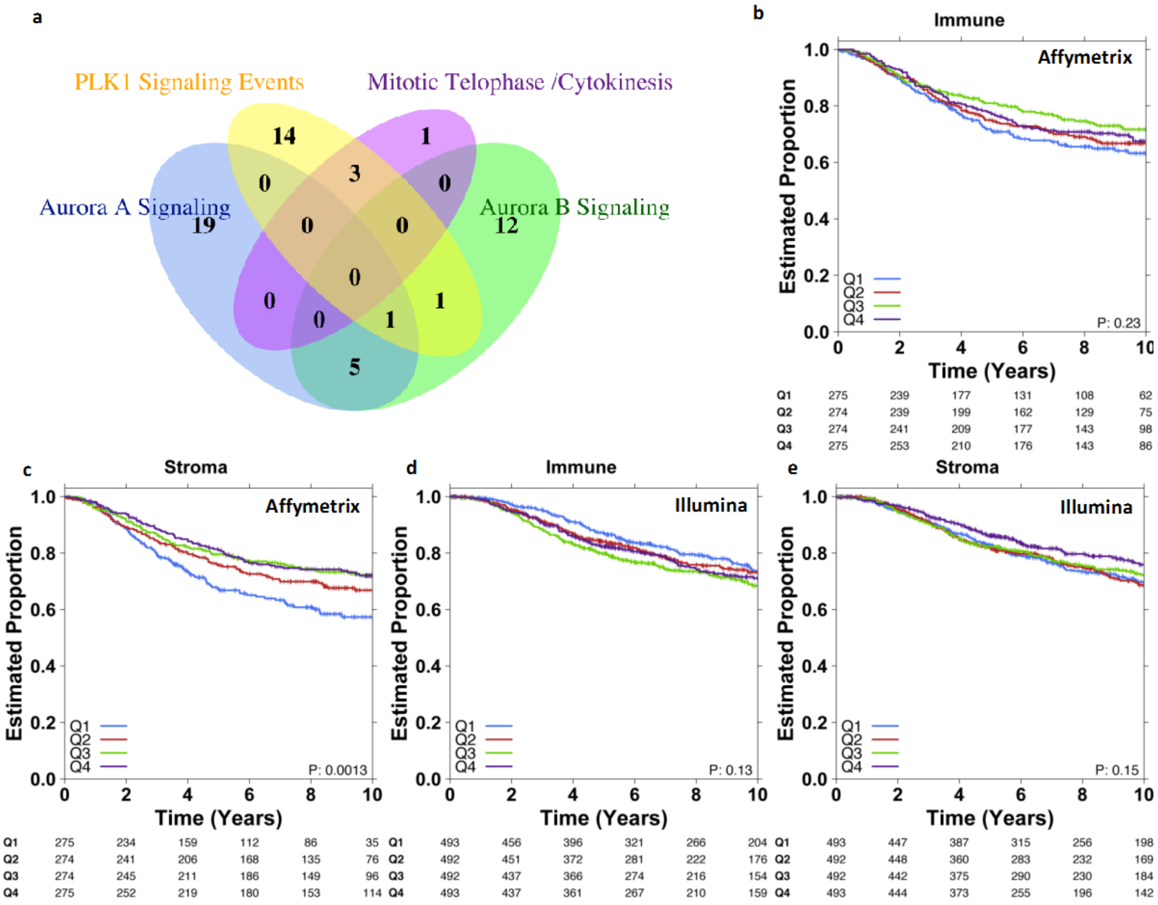

Supplementary Figure 10

Overlap of genes in cell cycle subnetwork modules, and prognostic assessment of immune and stromal scores. (a) Venn diagram showing overlapping genes between proliferation subnetwork modules derived from the pathways of Aurora A signaling (module 1), Aurora B signaling (module 1), *PLK1* signaling events (module 1) and Mitotic Telophase/Cytokinesis (module 1). The maximal overlap was of a single gene (*AURKA*) common across three modules (Aurora A, Aurora B and PLK1 modules). Module number in parenthesis refers to unique module number within a pathway in SIMMS' network database (SIMMS R package). **(b, c)** Prognostic assessment of Immuno and Stromal scores estimated using ESTIMATE in Affymetrix based breast cancer validation cohorts (**Supplementary Table 2**). **(d, e)** Prognostic assessment of Immuno and Stromal scores estimated using ESTIMATE in Illumina based Metabric breast cancer

cohort. For b-e, patient groups (Q1-Q4) were created using quantiles of Immuno/Stromal scores.

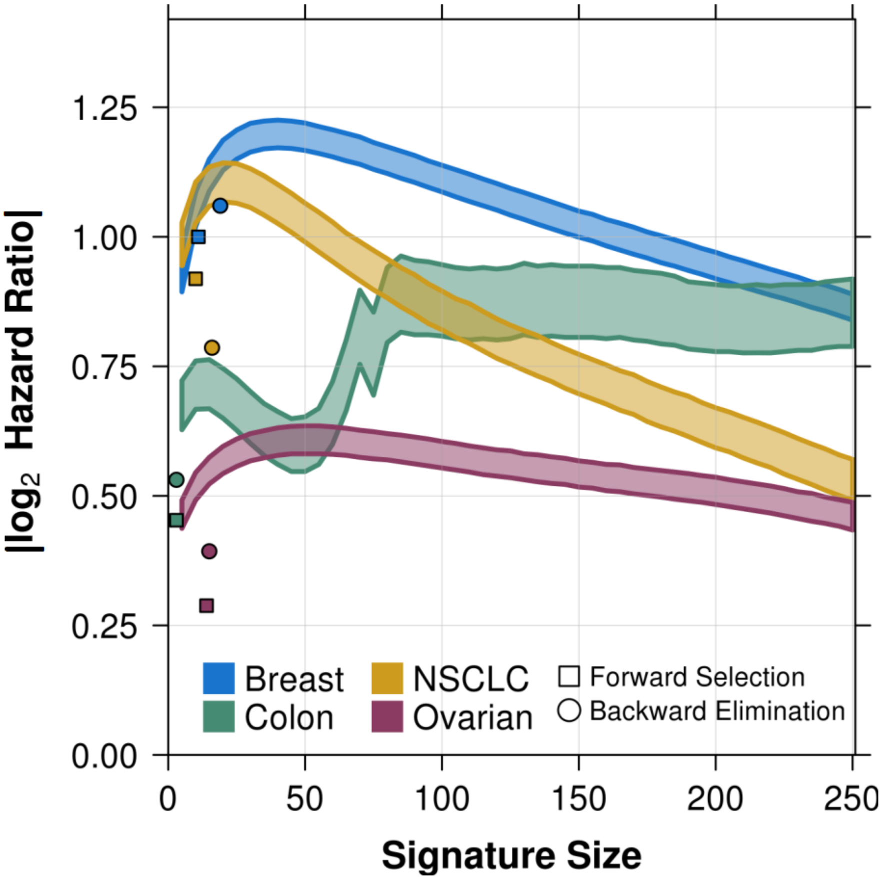

Supplementary Figure 11

Resampling of subnetworks database assessing sensitivity to initialisation size of SIMMS' multivariate models. Performance (SIMMS Model N) of breast, colon, NSCLC and ovarian cancer candidate biomarkers represented as a function of marker size. Jackknifing was performed over the subnetwork marker space for various tumour types. Ten million unique markers (200,000 for each marker size n=5,10,15,…,250) were randomly sampled using all 500 subnetworks regardless of their size. All biomarkers were generated using two independent machine learning paradigms; backward elimination and forward selection. The prognostic performance of each candidate biomarker was measured by taking the absolute value of the $\log_2$-transformed hazard ratio

estimated with a multivariate Cox proportional hazards model based on SIMMS Model N scores. These randomization results depict a range of prognostic performance between 75th and 95th percentiles at each marker size and were used as a guide to estimate the optimal top *n* number of subnetwork modules required to establish a multivariate classifier for a given tumour type.

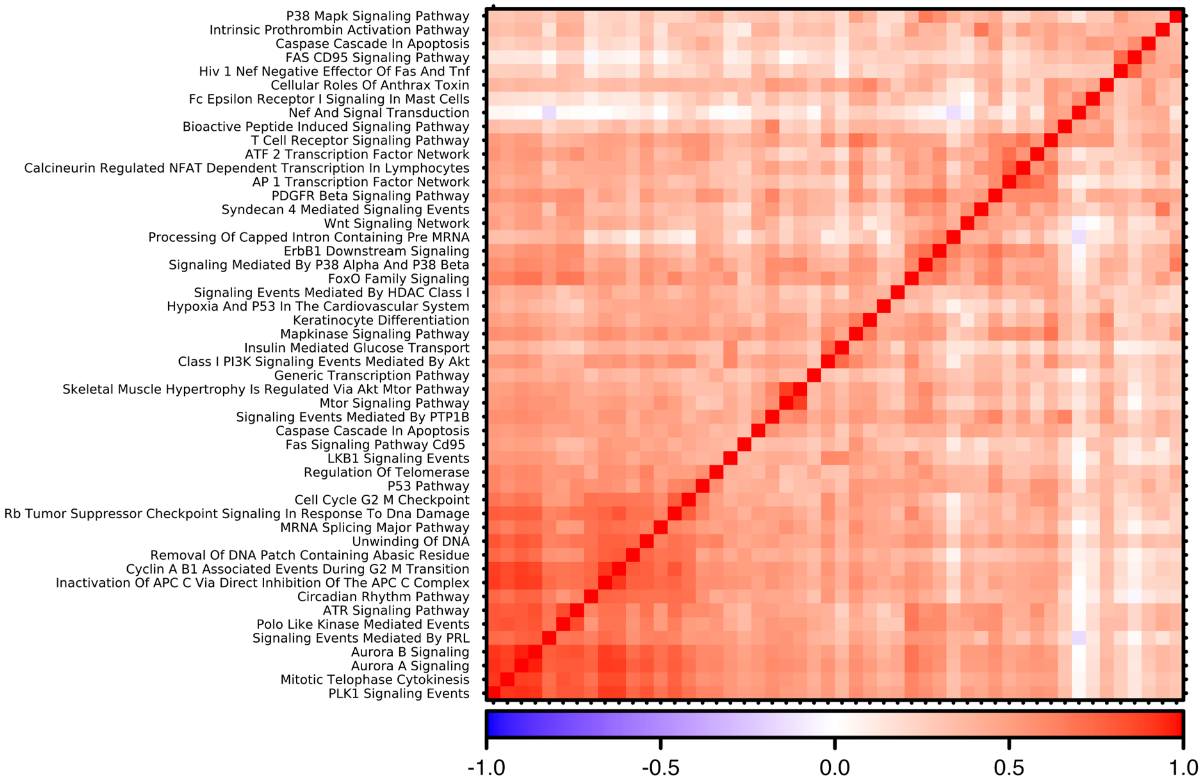

Supplementary Figure 12

Co-expression of subnetwork risk scores in breast cancer. Heatmap of correlation and cluster analysis of patient's risk score of top ranked 50 subnetwork modules of breast cancer (validation datasets only). The plot displays activity of subnetworks as well as clusters of highly co-expressed modules as indicated in dark red clusters.

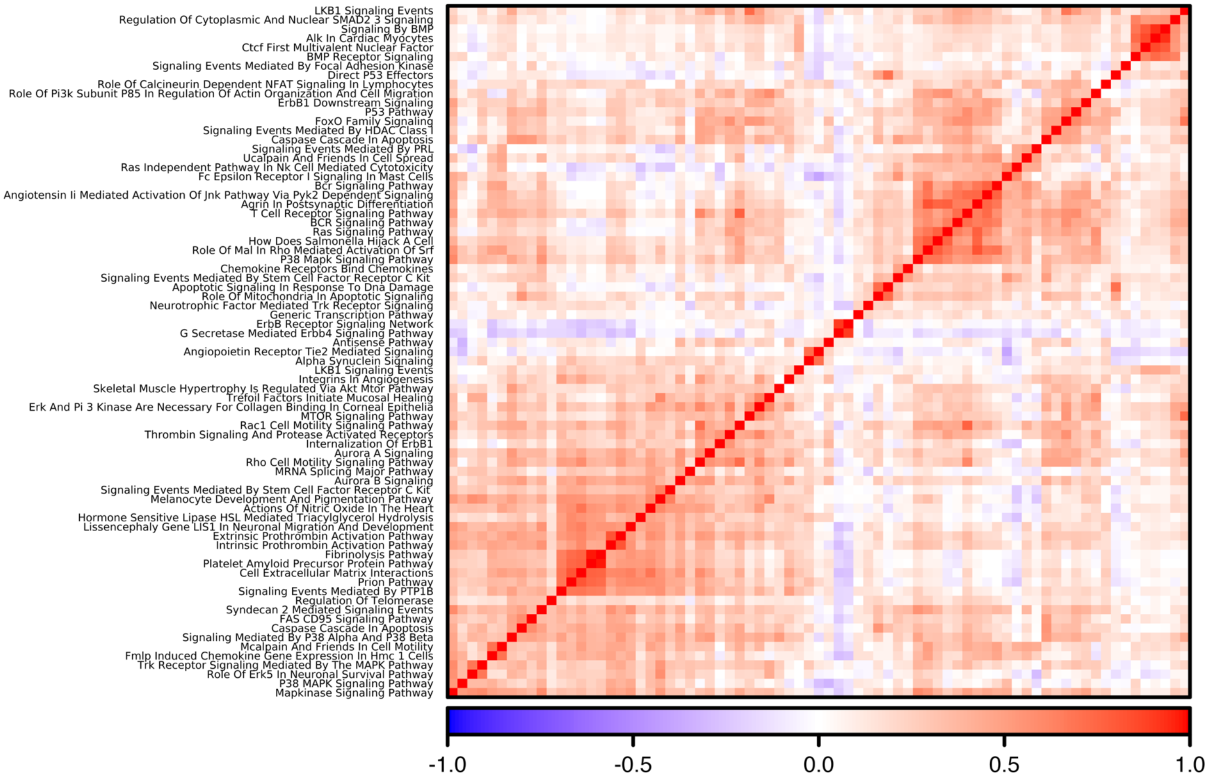

Supplementary Figure 13

Co-expression of subnetwork risk scores in colon cancer. Heatmap of correlation and cluster analysis of patients' risk score of top ranked 75 subnetwork modules of colon cancer (validation datasets only). The plot displays biological activity of subnetworks as well as clusters of highly co-expressed modules as indicated in dark red clusters.

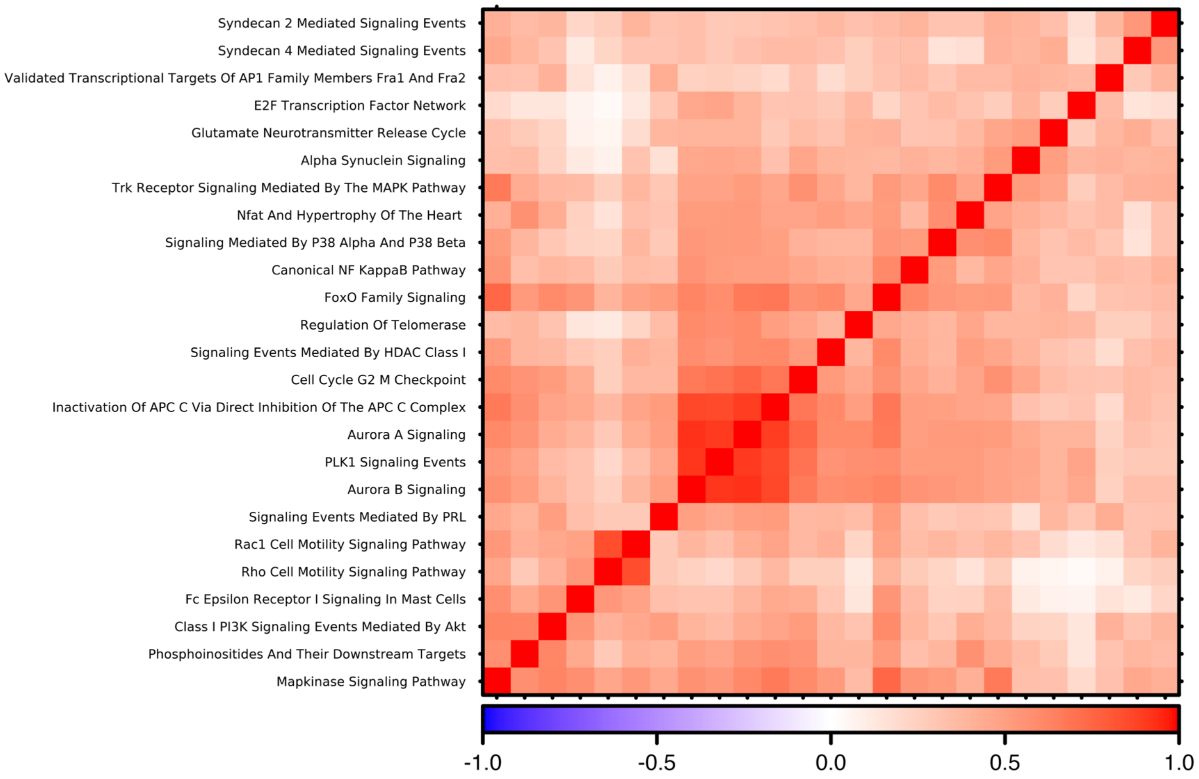

Supplementary Figure 14

Co-expression of subnetwork risk scores in NSCLC. Heatmap of correlation and cluster analysis of patients' risk score of top ranked 25 subnetwork modules of NSCLC (validation datasets only). The plot displays biological activity of subnetworks as well as clusters of highly co-expressed modules as indicated in dark red clusters.

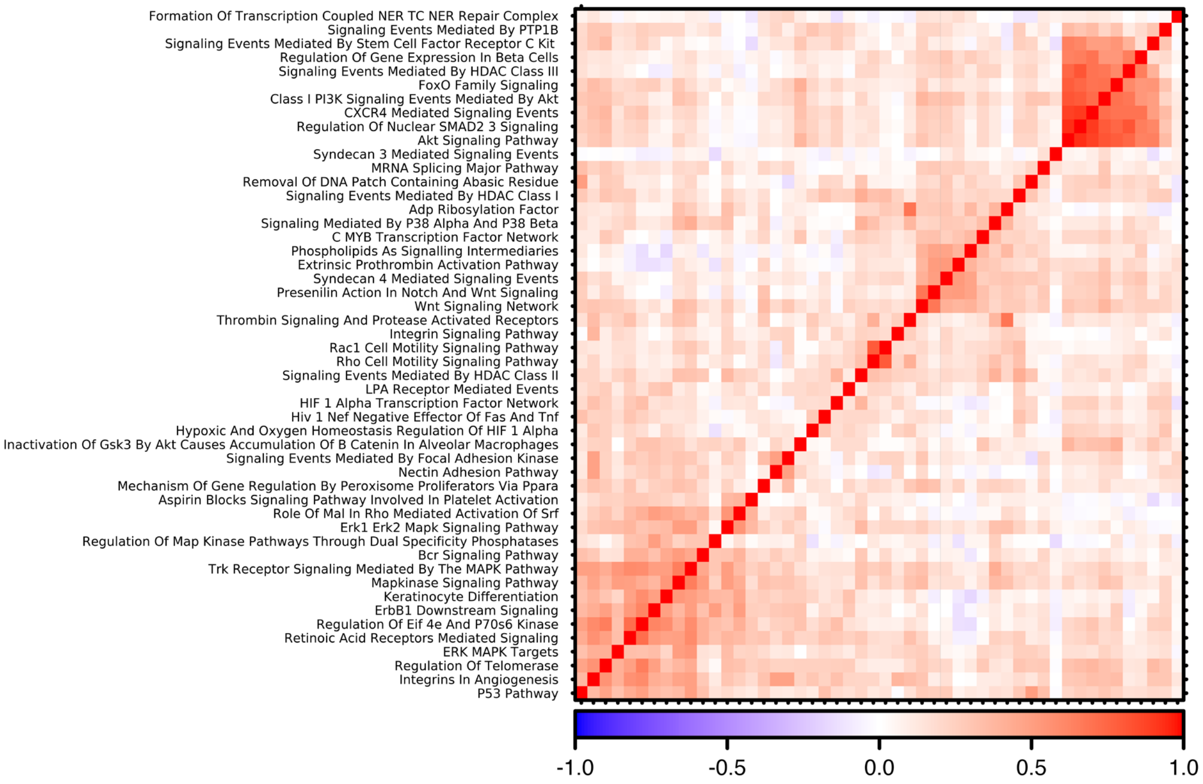

Supplementary Figure 15

Co-expression of subnetwork risk scores in ovarian cancer. Heatmap of correlation and cluster analysis of patients' risk score of top ranked 50 subnetwork modules of ovarian cancer (validation datasets only). The plot displays biological activity of subnetworks as well as clusters of highly co-expressed modules as indicated in dark red clusters.

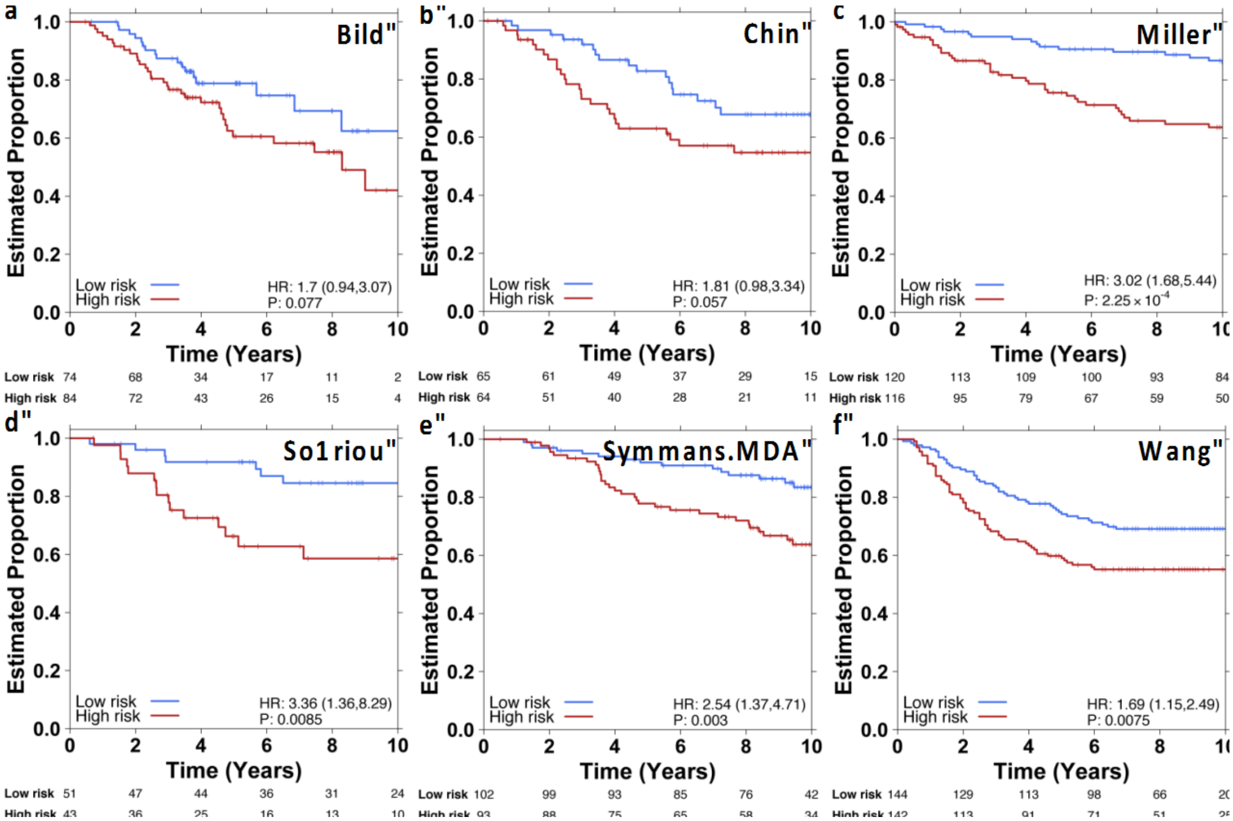

Supplementary Figure 16

Independent validation in breast cancer cohorts. Kaplan-Meier survival plots using SIMMS' Model N on 6 breast cancer validation sets (**Supplementary Table 2**) (10-year survival truncation) with subnetwork module selection performed through generalized linear models with *L1*-regularization (10-fold cross validation on training set). Model was initialised with the top ranked 50 subnetwork modules.

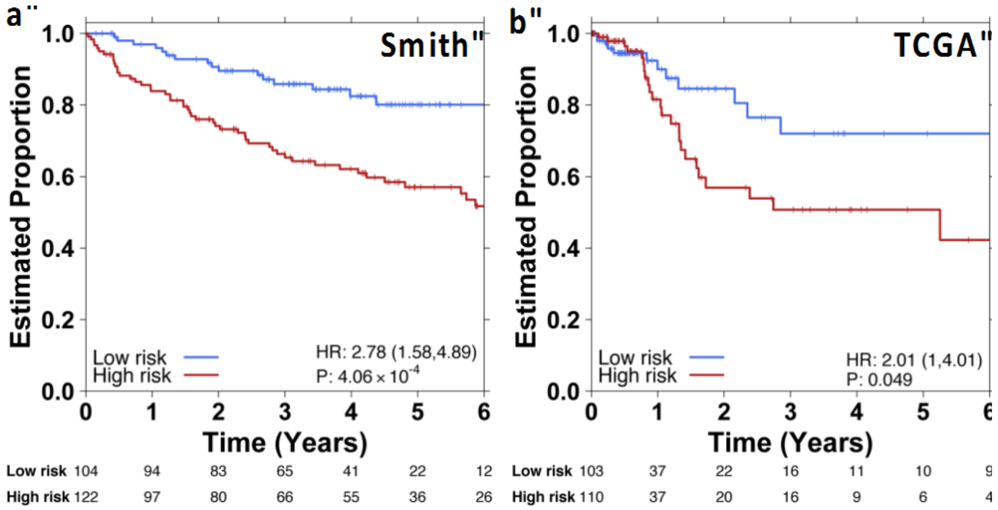

Supplementary Figure 17

Independent validation in colon cancer cohorts. Kaplan-Meier survival plots using SIMMS' Model N on 2 colon cancer validation sets (**Supplementary Table 3**) (6-year survival truncation) with subnetwork module selection performed through generalized linear models with *L1*-regularization (10-fold cross validation on training set). Model was initialised with the top ranked 75 subnetwork modules.

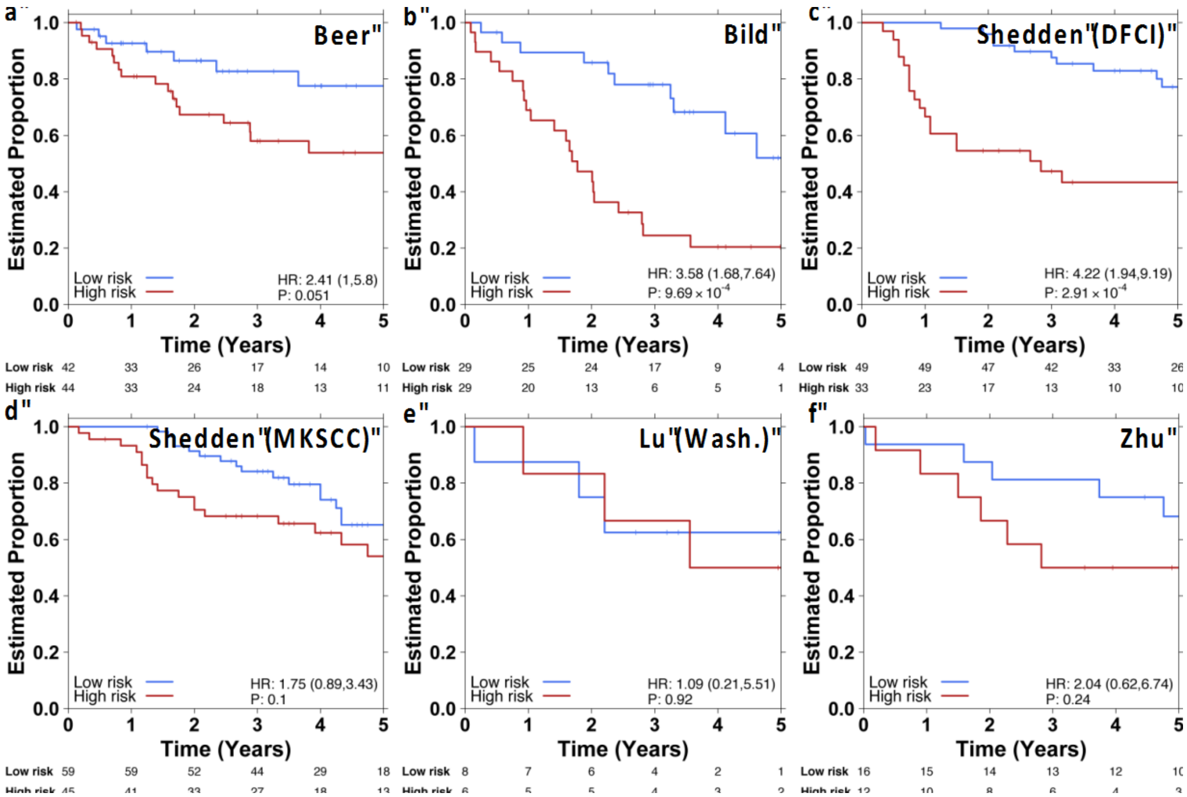

Supplementary Figure 18

Independent validation in NSCLC cohorts. Kaplan-Meier survival plots using SIMMS' Model N on 6 NSCLC validation sets (**Supplementary Table 4**) (5-year survival truncation) with subnetwork module selection performed through generalized linear models with *L1*-regularization (10-fold cross validation on training set). Model was initialised with the top ranked 25 subnetwork modules.

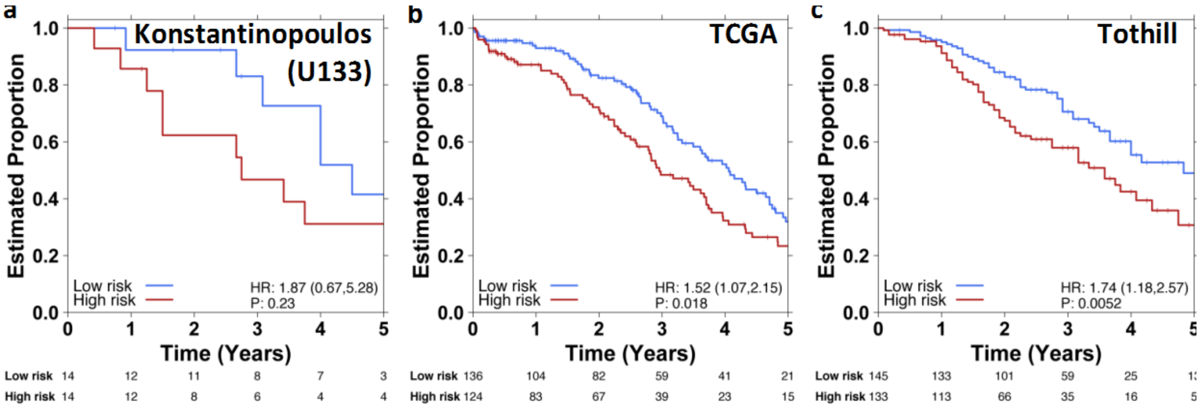

Supplementary Figure 19

Independent validation in ovarian cancer cohorts. Kaplan-Meier survival plots using SIMMS' Model N on 3 ovarian cancer validation sets (**Supplementary Table 5**) (5-year survival truncation) with subnetwork module selection performed through generalized linear models with *L1*-regularization (10-fold cross validation on training set). Model was initialised with the top ranked 50 subnetwork modules.

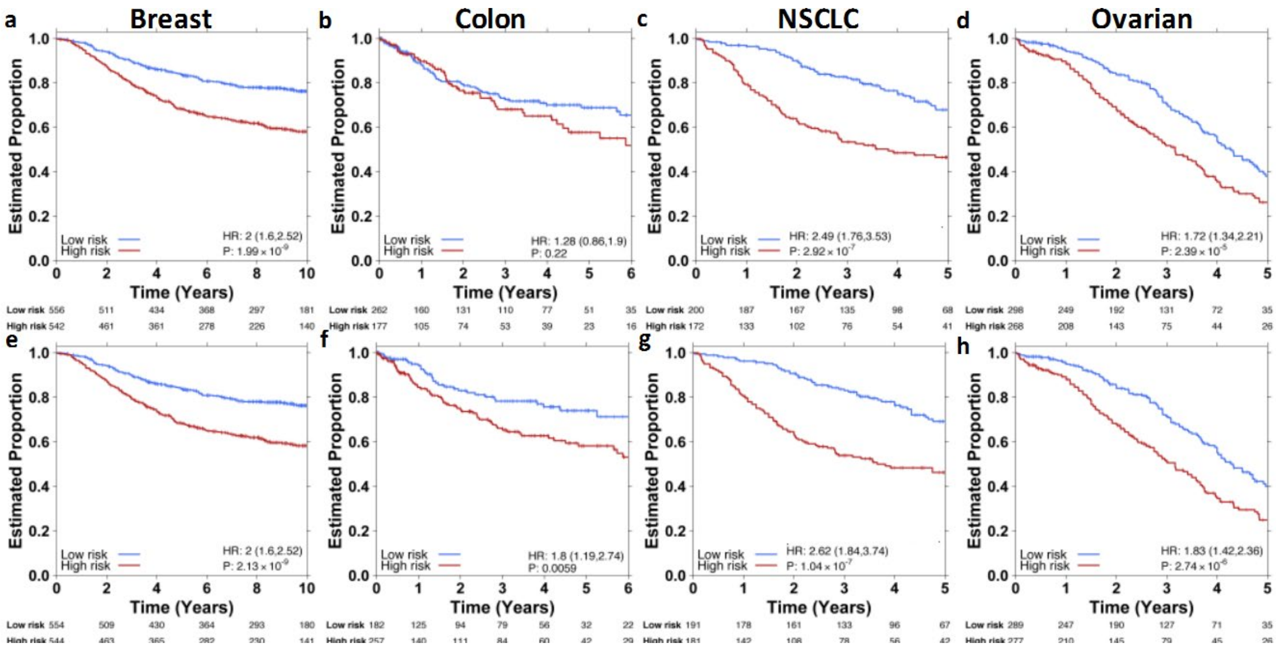

Supplementary Figure 20

Assessment of alternative machine learning algorithms. Kaplan-Meier survival plots of SIMMS' Model N in validation cohorts of various tumour types using alternative training algorithms; backwards elimination **(a-d)** and forward selection **(e-h)**.

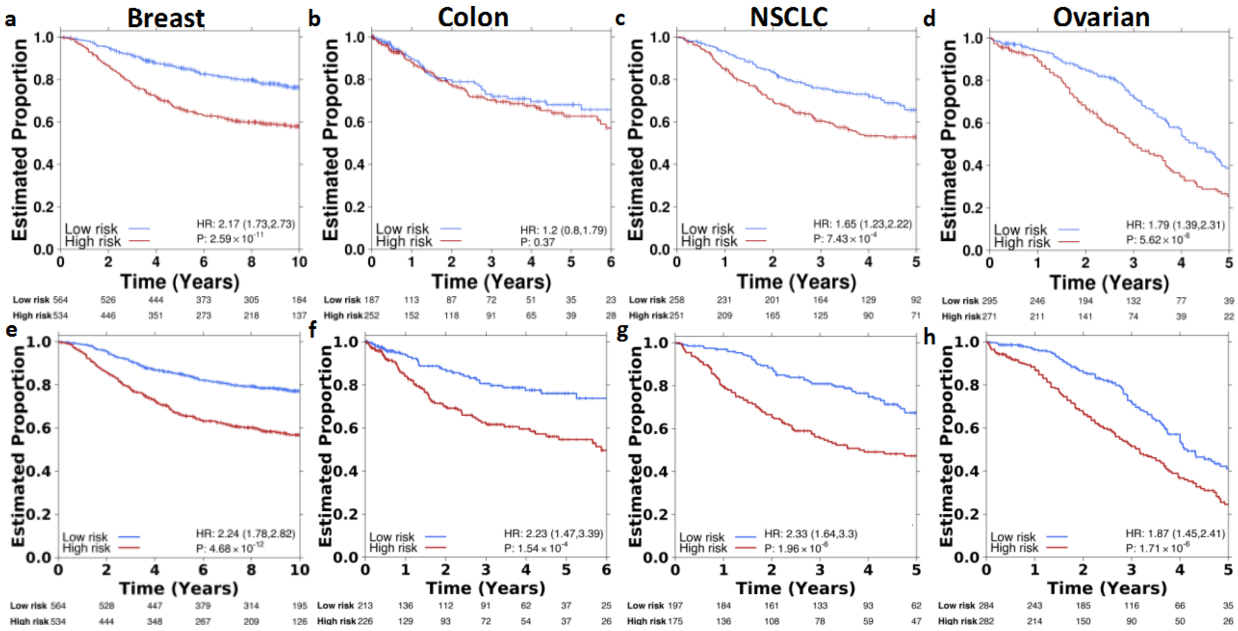

Supplementary Figure 21

Prognostic assessment of naïve and SIMMS model with all the genes in the subnetwork database. Kaplan-Meier survival plots of validation sets in each tumour type (**a-d**) for a Cox proportional hazard model using LASSO (*L1*-regularization) with all genes contained in any subnetwork as model variables. (**e-h**) Kaplan-Meier survival plots of validation sets in each tumour type for a Cox proportional hazard model fitted using risk scores estimated by SIMMS on a single module containing all the genes across all subnetworks.

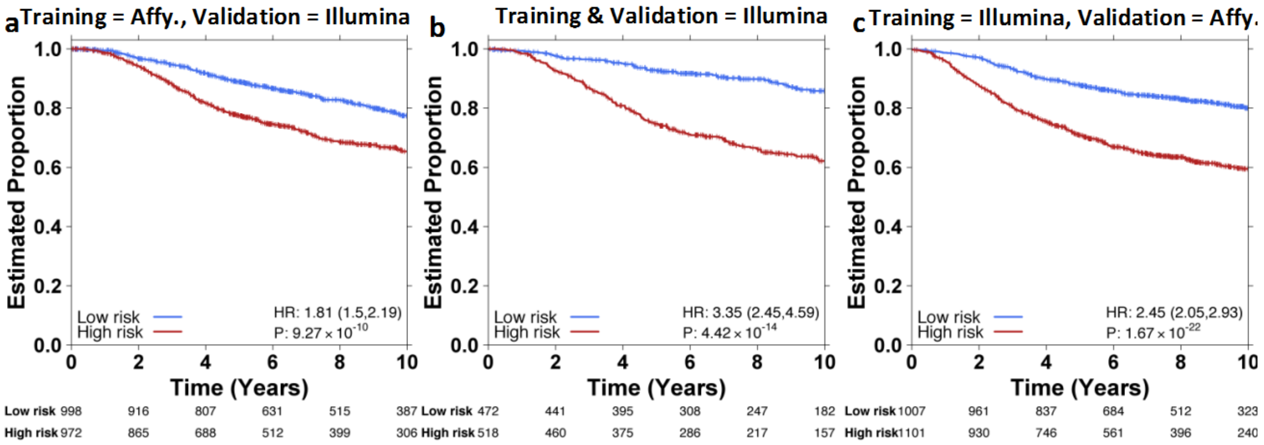

Supplementary Figure 22

Reproducibility of SIMMS' models across mRNA quantification platforms. Kaplan-Meier survival plots of SIMMS' Model N based predictions on the Metabric validation cohort. Separate classifiers were created using the Affymetrix based breast cancer training cohorts (**Supplementary Table 2**) and Illumina based breast cancer cohort (Metabric training set). These two classifiers were validated on Illumina based breast cancer cohort (Metabric validation set) **(a,b)** and Affymetrix based breast cancer validation cohorts, respectively **(c)**. All models were trained in 10-fold cross validation setting.

a"

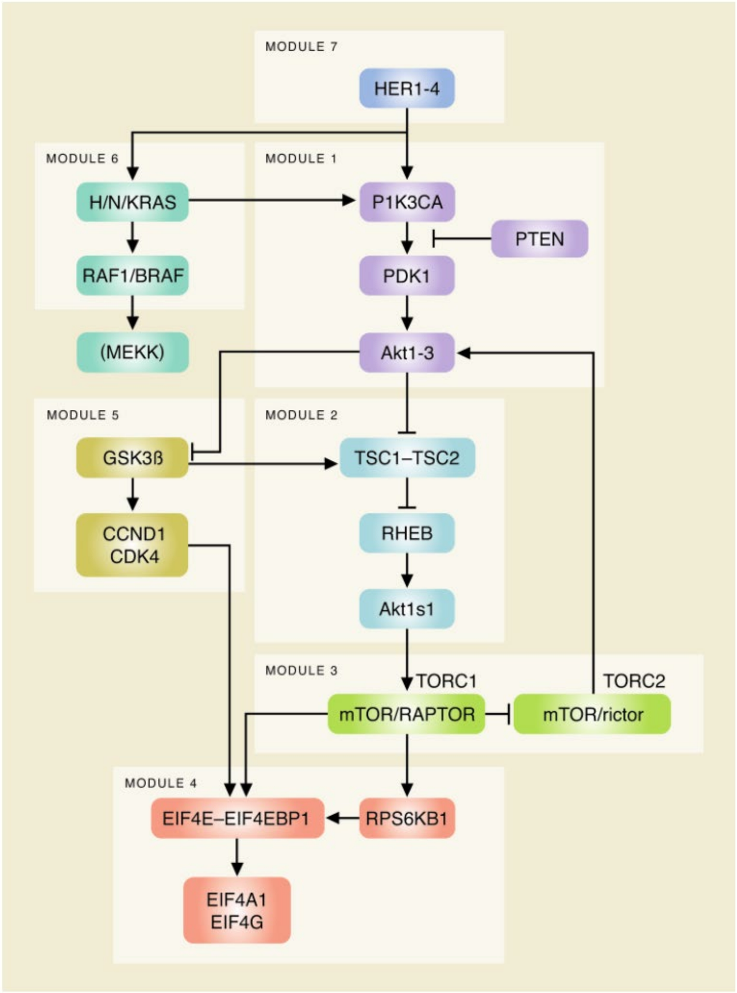

Supplementary Figure 23

Schematic overview of the PI3K signalling pathway. Figure illustrating key relationships between modules assessed in the current study. Modules 1-7 are highlighted with key signalling inter-relationships between the member genes.

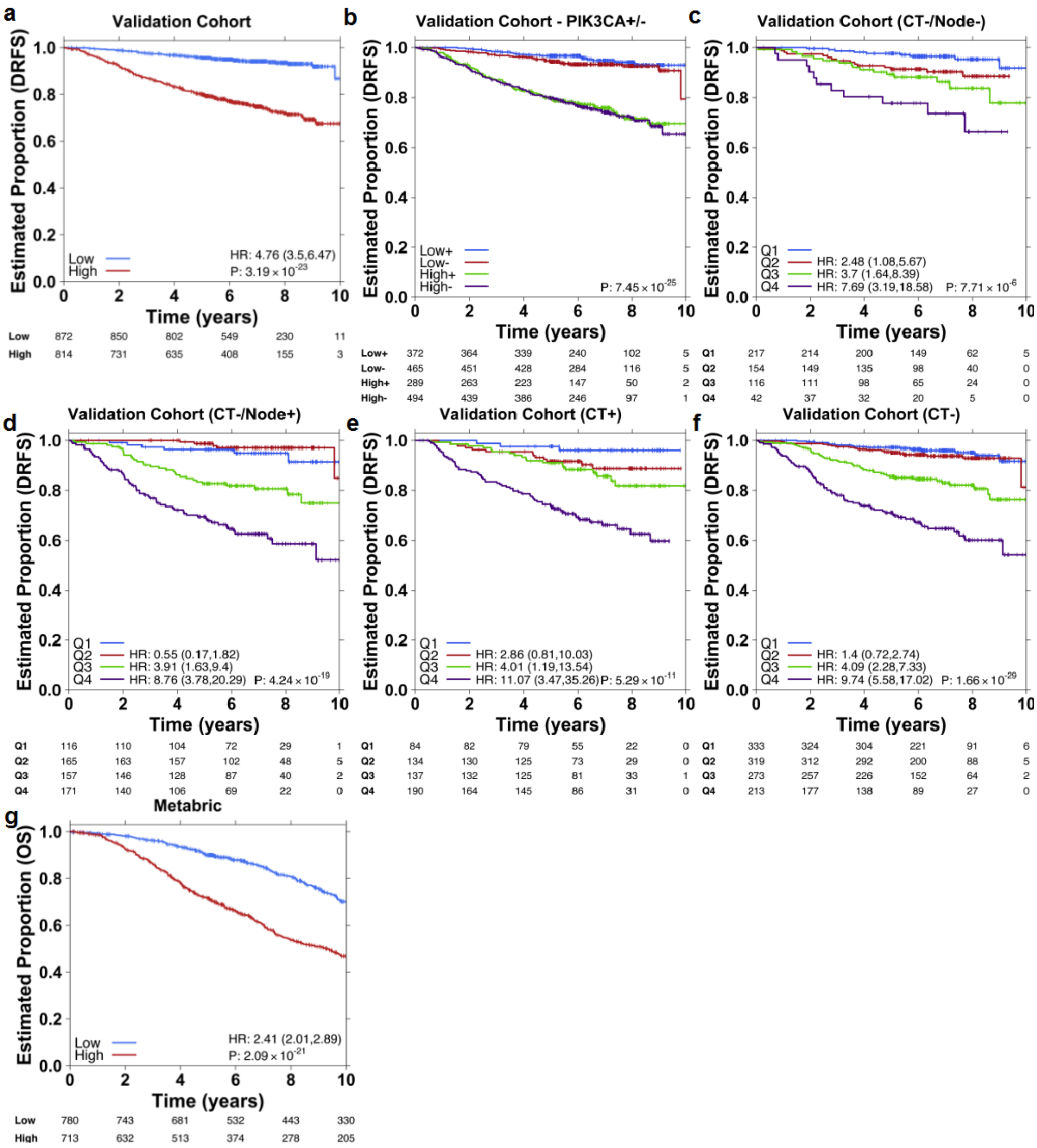

Supplementary Figure 24

Validation of SIMMS' PI3K risk predictor. (a) Prognostic assessment of SIMMS' PI3K risk predictor by median-dichotomizing predicted risk scores into low- and high-risk groups. **(b)** Prognostic assessment of model in (a) stratified by PIK3CA mutations. Patients were classified into low- and high-risk groups, and

each was further divided by PIK3CA mutant (+) and wild-type (-) status. **(c-d)** Prognostic assessment of PI3K predictor on patients which were not treated with chemotherapy and were further stratified into node –ve and node +ve groups. **(e, f)** Prognostic performance assessment in patients with- and without chemotherapy arms of the validation cohort. Within each subgroup, risk score quartiles Q2-Q4 were compared against Q1 using Cox proportional hazards modelling and the log-rank test**. (g)** Validation of SIMMS' PI3K risk predictor (FFPE samples trained model) on ER+ subset of Metabric cohort (fresh frozen samples). Risk scores of Metabric samples were dichotomised using median risk score derived from TEAM cohort.

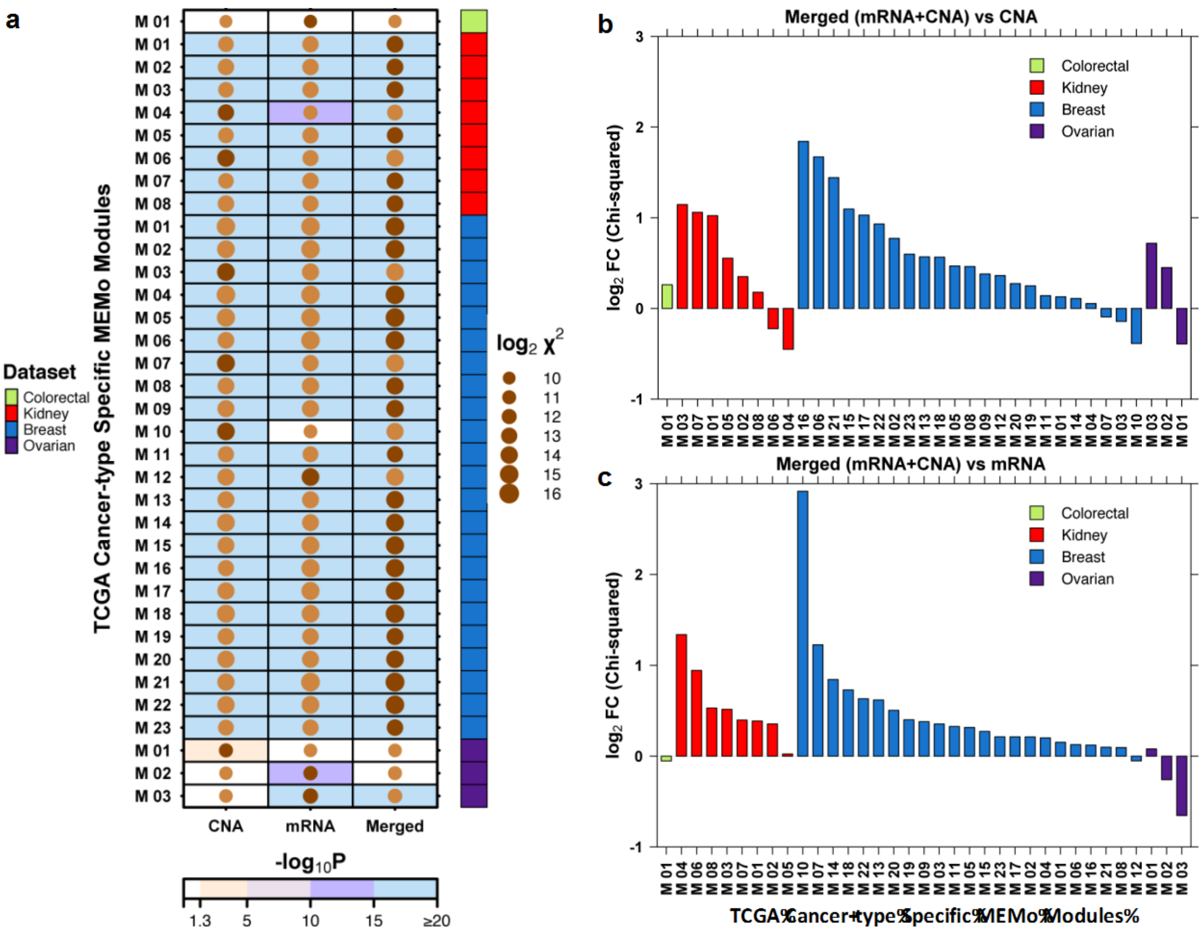

Supplementary Figure 25

Multi-modal assessment of SIMMS. Multi-modal prognostic biomarkers for breast, colon, kidney and ovarian cancers. **(a)** Dot plot of summarised (Fisher's combined probability test) chi-square estimates and P values for each of the MEMo derived cancer-type specific subnetwork modules (M*x*) (**Supplementary Methods section 5, Supplementary Table 21**). Covariates represent colours of each cancer type. Size of the dot represents log(chi-square) estimate resulting from the meta-analysis of Cox P values (1000 random subsets for each profile in each cancer type). A Cox proportional hazards model was fitted to dichotomous risk scores across the entire validation cohort to assess survival association of predicted risk groups. Crosses represent absence of a module from a particular cancer type. **(b, c)** Performance comparison of multi-modal prognostic models

(Merged mRNA+CNA) against CNA models (b) and mRNA models (c) in each cancer type using MEMo modules of that particular cancer. Within each cancer type, modules are sorted by the largest fold-change in chi-squared values; with positive values indicating improved prognostication by the multi-modal model over CNA or mRNA models.

Supplementary References

1. Breitling, R., Armengaud, P., Amtmann, A. & Herzyk, P. Rank products: a simple, yet powerful, new method to detect differentially regulated genes in replicated microarray experiments. *FEBS Lett* **573**, 83-92 (2004).
2. Sotiriou, C. et al. Gene expression profiling in breast cancer: understanding the molecular basis of histologic grade to improve prognosis. *J Natl Cancer Inst* **98**, 262-272 (2006).
3. Symmans, W.F. et al. Genomic index of sensitivity to endocrine therapy for breast cancer. *J Clin Oncol* **28**, 4111-4119 (2010).
4. Lee, E., Chuang, H.Y., Kim, J.W., Ideker, T. & Lee, D. Inferring pathway activity toward precise disease classification. *PLoS Comput Biol* **4**, e1000217 (2008).
5. Guo, Z. et al. Towards precise classification of cancers based on robust gene functional expression profiles. *BMC Bioinformatics* **6**, 58 (2005).
6. Bild, A.H. et al. Oncogenic pathway signatures in human cancers as a guide to targeted therapies. *Nature* **439**, 353-357 (2006).
7. Bueno, R. et al. Comprehensive genomic analysis of malignant pleural mesothelioma identifies recurrent mutations, gene fusions and splicing alterations. *Nat Genet* **48**, 407-416 (2016).
8. Zhao, X. et al. Systematic assessment of prognostic gene signatures for breast cancer shows distinct influence of time and ER status. *BMC Cancer* **14**, 211 (2014).
9. Oh, S.C. et al. Prognostic gene expression signature associated with two molecularly distinct subtypes of colorectal cancer. *Gut* **61**, 1291-1298 (2012).
10. Smith, J.J. et al. Experimentally derived metastasis gene expression profile predicts recurrence and death in patients with colon cancer. *Gastroenterology* **138**, 958-968 (2010).
11. Chen, H.Y. et al. A five-gene signature and clinical outcome in non-small-cell lung cancer. *The New England journal of medicine* **356**, 11-20 (2007).
12. Lau, S.K. et al. Three-gene prognostic classifier for early-stage non small-cell lung cancer. *Journal of clinical oncology : official journal of the American Society of Clinical Oncology* **25**, 5562-5569 (2007).
13. Shedden, K. et al. Gene expression-based survival prediction in lung adenocarcinoma: a multi-site, blinded validation study. *Nature medicine* **14**, 822-827 (2008).
14. Boutros, P.C. et al. Prognostic gene signatures for non-small-cell lung cancer. *Proceedings of the National Academy of Sciences of the United States of America* **106**, 2824-2828 (2009).
15. Starmans, M.H. et al. Exploiting the noise: improving biomarkers with ensembles of data analysis methodologies. *Genome Med* **4**, 84 (2012).
16. Yoshihara, K. et al. High-risk ovarian cancer based on 126-gene expression signature is uniquely characterized by downregulation of antigen

presentation pathway. *Clinical cancer research : an official journal of the American Association for Cancer Research* **18**, 1374-1385 (2012).

17. The Cancer Genome Atlas Research Network Integrated genomic analyses of ovarian carcinoma. *Nature* **474**, 609-615 (2011).
18. Mankoo, P.K., Shen, R., Schultz, N., Levine, D.A. & Sander, C. Time to recurrence and survival in serous ovarian tumors predicted from integrated genomic profiles. *PLoS One* **6**, e24709 (2011).
19. Wu, G. & Stein, L. A network module-based method for identifying cancer prognostic signatures. *Genome biology* **13**, R112 (2012).
20. Waggott, D. et al. NanoStringNorm: an extensible R package for the pre-processing of NanoString mRNA and miRNA data. *Bioinformatics* **28**, 1546-1548 (2012).
21. Sabine, V.S. et al. Mutational analysis of PI3K/AKT signaling pathway in tamoxifen exemestane adjuvant multinational pathology study. *J Clin Oncol* **32**, 2951-2958 (2014).
22. Cuzick, J. et al. Prognostic value of a combined estrogen receptor, progesterone receptor, Ki-67, and human epidermal growth factor receptor 2 immunohistochemical score and comparison with the Genomic Health recurrence score in early breast cancer. *J Clin Oncol* **29**, 4273-4278 (2011).
23. The Cancer Genome Atlas Research Network Comprehensive genomic characterization defines human glioblastoma genes and core pathways. *Nature* **455**, 1061-1068 (2008).
24. Ciriello, G., Cerami, E., Aksoy, B.A., Sander, C. & Schultz, N. Using MEMo to discover mutual exclusivity modules in cancer. *Curr Protoc Bioinformatics* **Chapter 8**, Unit 8 17 (2013).
25. Network, T.C.G.A. Comprehensive molecular portraits of human breast tumours. *Nature* **490**, 61-70 (2012).
26. Cancer Genome Atlas Research, N. Comprehensive molecular characterization of clear cell renal cell carcinoma. *Nature* **499**, 43-49 (2013).
27. The Cancer Genome Atlas Research Network Comprehensive molecular characterization of human colon and rectal cancer. *Nature* **487**, 330-337 (2012).
