# Supplementary material for: Pathway-based subnetworks enable cross-disease biomarker discovery
Source: Nat Commun. 2018 Nov 12;9:4746. doi: 10.1038/s41467-018-07021-3 (PMC6232113; doi:10.1038/s41467-018-07021-3)
Supplement: Supplementary file 2 — Description of Additional Supplementary files [file 41467_2018_7021_MOESM2_ESM.pdf]

Supplementary Data Legends

Supplementary Table 1

Overview of pathways extracted from NCI-Nature pathway interaction database, which is an amalgamation of NCI-curated, Reactome and BioCarta pathways databases. Protein-protein interaction subnetworks were extracted to define subnetwork modules and subsequently used to project molecular profiles of cancer patients.

Supplementary Table 2

List of breast cancer studies included in preliminary analysis[1-13]. Li et al. and Loi et al. were regarded as outliers following univariate analyses (**Supplementary Methods section 1, Supplementary Fig. 3**), and subsequently removed from further analyses. The remaining studies were divided into two groups to keep a modest balance in the size and platform distribution for training and testing of prognostic models.

Supplementary Table 3-5

List of colon[14-17], NSCLC[1, 18-22] and ovarian[1, 23-27] cancer studies used for training and validation of prognostic models using SIMMS. Studies within each cancer type were divided into training and independent validation cohorts maintaining homogeneity in size and platforms, and in part further addressed through 10-fold cross validation and permutation analyses. Datasets referred to as colon cancer cohorts in this study contained both colon and colorectal cancer patients.

Supplementary Table 6

Hazard ratios (95% CI, P values, size of the validation cohort and q values; Benjamini & Hochberg method) of patients' risk score based classification in breast cancer. *a, b and c* represent Model N+E (nodes and interactions/edges), Model N (nodes only) and Model E (interactions/edges only) respectively. A univariate Cox proportional hazards model was fitted to each of the subnetwork

markers and subsequently applied to predict patient risk score in the validation cohort. The survival differences between the median-dichtomised risk scores (low and high-risk groups) were assessed using Kaplan-Meier analysis. Table 6d shows univariate Cox model coefficients (log2(HR)) of genes involved in T-cell receptor signalling subnetwork, which were used to estimate per patient risk score. Genes highlighted in green (Wald-test P < 0.5) were selected to contribute toward risk score estimation.

Supplementary Tables 7-9

Hazard ratios (95% CI, P values, size of the validation cohort and q values; Benjamini & Hochberg method) of patients' risk score based classification in Colon, NSCLC and Ovarian cancers. *a, b and c* represent Model N+E (nodes and interactions/edges), Model N (nodes only) and Model E (interactions/edges only) respectively. A univariate Cox proportional hazards model was fitted to each of the subnetwork markers (**Table 7a-c: Colon, Table 8a-c: NSCLC and Table 9a-c: Ovarian**) and subsequently applied to predict patient risk score in the validation cohort. The survival differences between the median-dichtomised risk scores (low and high-risk groups) were assessed using Kaplan-Meier analysis. **Table 9d** contains co-occurrence analysis of platinum responders/non-responders and SIMMS predicted risk groups in TCGA ovarian cancer cohort. Previously published data on treatment response was used[28].

Supplementary Tables 10-13

List of subnetwork modules following feature selection performed through Cox model using generalized linear models with LASSO (*L1*-regularization) in breast (**Table 10**), colon (**Table 11**), NSCLC (**Table 12**), and ovarian (**Table 13**). Each table contains selected subnetwork modules in the final model along with coefficients (beta) of the fit. All models were trained in 10-fold cross validation setting. Subnetwork modules were scored using SIMMS' Model N.

Supplementary Table 14

Comparison of previously published breast, colon, NSCLC and ovarian cancer biomarkers with the SIMMS' identified markers. Cox model HR (95% CI) and P values (Wald-test or Logrank-test where appropriate) are shown for all the subnetwork models. Only P value is reported when the HR (95% CI) was not available in the original study. For fair comparison, we focussed on those biomarkers which shared a validation cohort/s with SIMMS' validation cohorts, except for Smith *et al.* colon cancer dataset, which was partly used as the training set in the original biomarker while completely used as a validation set by the SIMMS colon cancer classifier. Highlighted in pink are the Cox statistics by the best performing classifier for each validation dataset.

Supplementary Table 15

REMARK[29] information of team study describing total number of samples recruited, eligible and analysed.

Supplementary Table 16

Univariate prognostic assessment of mRNA abundance profiles. mRNA abundance profiles in TEAM training cohort were median-dichotomized into low- and high-risk groups except for ERBB2 (HER2). ERBB2 dichotomization was performed using Expectation-maximization clustering. DRFS was used as the survival end point. Cox proportional hazards model was used to estimate the Hazard ratios followed by the Wald-test for the significance of difference between the risk groups. P values were corrected for multiple comparisons using Benjamini & Hochberg method. The varying *n* cohorts is an artefact of normalisation/log_2 transformation of zero (0 abundance) for some patients.

Supplementary Table 17

Univariate prognostic assessment of clinical variables and mutational profiles in TEAM training cohort. DRFS was used as the survival end point. Cox proportional hazards model was used to estimate the Hazard ratios. The significance of association between DRFS and dichotomous variables (age,

HER2 status, and mutational profiles) was assessed using the Wald-test. However, Log-rank test was used for multi-category variables (grade, T-stage and N-stage). Prognostic assessment of grade and stage was conducted such that the grade 2 and 3 patients were compared against the baseline grade 1; N Stage 1, 2 and 3 were compared against N Stage 0 (node-negative); and T Stage 2 and 3 were compared against the baseline T Stage 1. For mutational profiles, wild-type carriers were compared against mutated samples for a given gene (HER2, *PIK3CA, AKT1* and *RAS*).

Supplementary Table 18

List of PIK3CA pathway modules and corresponding genes. Modules were derived on the basis of underlying biological functionality.

Supplementary Table 19

Multivariate PIK3CA modules-derived prognostic model. Model parameters were estimated using a multivariate Cox proportional hazards model initialized with eight mRNA modules (**Supplementary Table 18**), age, grade, pathological size and N-stage. Model was further refined using backwards elimination resulting in the variables presented in this table. The model was trained using TEAM training cohort. The refined model was subsequently used to predict patient risk score in the TEAM validation cohort. Survival differences between the median-dichotomized risk scores as well as quartiles of the risk scores were assessed using Kaplan-Meier analysis.

Supplementary Table 20

Distribution of patients' tumour and clinical characteristics in randomly assigned Training and Validation cohorts. Numbers in the parentheses indicate relative proportion within each group. Unequal distribution of patient characteristics

across randomly assigned Training and Validation cohorts was tested using Fisher's exact test followed by adjustment of probability values for multiple comparisons. Patients within the pathology research study were matched to the overall TEAM trial cohort, see previous publication[30].

Supplementary Table 21

List of modules curated from TCGA MEMo analysis in breast (BRCA), colorectal (COADREAD), kidney (KIRC) and ovarian (OV) cancers[17, 26, 31, 32]. These modules were used for creating multi-modal biomarkers.
